# SPTBN1 inhibits inflammatory responses and hepatocarcinogenesis via the stabilization of SOCS1 and downregulation of p65 in hepatocellular carcinoma

**DOI:** 10.7150/thno.49819

**Published:** 2021-02-20

**Authors:** Ling Lin, Shuyi Chen, Hua Wang, Bin Gao, Bhaskar Kallakury, Krithika Bhuvaneshwar, Katherine Cahn, Yuriy Gusev, Xue Wang, Yunan Wu, John L. Marshall, Xiuling Zhi, Aiwu Ruth He

**Affiliations:** 1 Department of Medicine and Oncology, Lombardi Comprehensive Cancer Center, Georgetown University, Washington, DC, USA.; 2 Department of Physiology and Pathophysiology, School of Basic Medical Sciences, Fudan University, Shanghai, China.; 3 Laboratory of Liver Diseases, National Institute on Alcohol Abuse and Alcoholism, National Institutes of Health, Bethesda, MD, USA.; 4 Innovation Center for Biomedical Informatics, Lombardi Comprehensive Cancer Center, Georgetown University, Washington, DC, USA.; 5 Department of Pathology, Ruijin Hospital, Shanghai Jiao Tong University School of Medicine, Shanghai, China.

**Keywords:** SPTBN1, pro-inflammatory cytokines, NF-κB, protein stabilization, SOCS1

## Abstract

**Background:** Spectrin, beta, non-erythrocytic 1 (SPTBN1), an adapter protein for transforming growth factor beta (TGF-β) signaling, is recognized as a tumor suppressor in the development of hepatocellular carcinoma (HCC); however, the underlying molecular mechanisms of this tumor suppression remain obscure.

**Methods:** The effects on expression of pro-inflammatory cytokines upon the inhibition or impairment of SPTBN1 in HCC cell lines and liver tissues of *Sptbn1^+/-^* and wild-type (WT) mice were assessed by analyses of quantitative real-time reverse-transcription polymerase chain reaction (QRT-PCR), enzyme linked immunosorbent assay (ELISA), Western blotting and gene array databases from HCC patients. We investigated the detailed molecular mechanisms underlying the inflammatory responses by immunoprecipitation-Western blotting, luciferase reporter assay, chromatin immunoprecipitation quantitative real time PCR (ChIP-qPCR), immunohistochemistry (IHC) and electrophoretic mobility shift assay (EMSA). The proportion of myeloid-derived suppressor cells in liver, spleen, bone marrow and peripheral blood samples from WT and *Sptbn1^+/-^* mice were measured by fluorescence-activated cell sorting (FACS) analysis. Further, the hepatocacinogenesis and its correlation with inflammatory microenvironment by loss of SPTBN1/SOCS1 and induction of p65 were analyzed by treating WT and *Sptbn1^+/-^* mice with 3,5-diethoxycarbonyl-1,4-dihydrocollidine (DDC).

**Results:** Loss of SPTBN1 in HCC cells upregulated the expression of pro-inflammatory cytokines including interleukin-1α (IL-1α), IL-1β, and IL-6, and enhanced NF-κB transcriptional activation. Mechanistic analyses revealed that knockdown of SPTBN1 by siRNA downregulated the expression of suppressor of cytokine signaling 1 (SOCS1), an E3 ligase of p65, and subsequently upregulated p65 accumulation in the nucleus of HCC cells. Restoration of SOCS1 abrogated this SPTBN1 loss-associated elevation of p65 in HCC cells. In human HCC tissues, SPTBN1 gene expression was inversely correlated with gene expression of IL-1α, IL-1β and IL-6. Furthermore, a decrease in the levels of SPTBN1 gene, as well as an increase in the gene expression of IL-1β or IL-6 predicted shorter relapse free survival in HCC patients, and that HCC patients with low expression of SPTBN1 or SOCS1 protein is associated with poor survival. Heterozygous loss of SPTBN1 (*Sptbn1^+/-^*) in mice markedly upregulated hepatic expression of IL-1α, IL-1β and IL-6, and elevated the proportion of myeloid-derived suppressor cells (MDSCs) and CD4^+^CD25^+^Foxp3^+^ regulatory T cells (Foxp3^+^Treg) cells in the liver, promoting hepatocarcinogenesis of mouse fed by DDC.

**Conclusions**: Our findings provided evidence that loss of SPTBN1 in HCC cells increases p65 protein stability via the inhibition of SOCS1 and enhances NF-κB activation, stimulating the release of inflammatory cytokines, which are critical molecular mechanisms for the loss of SPTBN1-induced liver cancer formation. Reduced SPTBN1 and SOCS1 predict poor outcome in HCC patients.

## Introduction

Spectrin, beta, non-erythrocytic 1 (SPTBN1) is an actin crosslinking and scaffold protein that is important for determination of cell shape, arrangement of functionally distinct transmembrane proteins, regulation of endocytic traffic, protein sorting, and development of a polarized differentiated epithelial cell 1-3. Homozygous mutant of SPTBN1 in mouse is embryonic lethal, suggesting that SPTBN1 may potentially play an essential role in embryonic development. As an adapter of SMAD3, SPTBN1 plays a pivotal role in facilitating TGF-β signal transduction 4, 5. Reduced expression of SPTBN1 was observed in many types of human cancers including hepatocellular carcinoma (HCC) and lung cancer 6, 7. Impairment or suppression of SPTBN1 in liver tissues of *Sptbn1^+/-^* mice and HCC cells promotes the expression of oncoproteins including cyclin D1, cyclin-dependent kinase 4 (Cdk4), c-Myc, MDM2, signal transducer and activator of transcription 3 (STAT3) and vimentin as well as stem cell markers such as EpCAM, Claudin7 and Oct4, and enhances oncogenic potential as characterized by increased anchorage-independent cell growth and xenograft tumor development, suggesting that SPTBN1 is a prominent suppressor of tumorigenesis 6-10.

From the analysis of HCC gene expression databases, we found that the suppression of *SPTBN1* is inversely correlated with the expression of inflammatory cytokines, including *interleukin-1α* (*IL-1α*)*, IL-1β*, and *IL-6*. Accumulating evidence suggest that IL-1α, IL-1β and IL-6 are cytokines that are involved in the formation of an inflammatory microenvironment and have been shown to participate in the initiation and progression of carcinogenesis, and also promote angiogenesis and stimulate the growth, invasion, and metastasis of tumor cells 11. IL-1α, IL-1β, and IL-6 are all down-stream targets of NF-κB through that NF-κB induces transcription of these cytokines via two NF-κB-like binding sites in the *IL-1α* promoter, one consensus NF-κB binding site and one nonconsensus CRE-like site in the *IL-1β* promoter, and one putative NF-κB binding site in the *IL-6* promoter 12-14.

NF-κB/Rel proteins are a family of transcription factors that regulate genes in the modulation of innate and adaptive immune responses. They serve as a critical link between inflammation and cancer formation, and have been shown to promote cancer progression via regulation of apoptosis, tumor angiogenesis, tumor invasion, and immune evasion 15-19. The human NF-κB family consists of five DNA binding subunits, including p65/RelA, c-Rel, RelB, NF-κB1 (p50/p105) and NF-κB2 (p52/p100). These proteins function as heterodimeric or homodimeric transcription factors of various NF-κB proteins to control the transcription of genes containing a decameric cis-acting κB binding site 16, 20. Activation of the NF-κB signaling pathway is induced by proinflammatory cytokines, lipopolysaccharides, growth factors, and activation of antigen receptors, followed by phosphorylation and ubiquitin-mediated proteolysis of IκB proteins, which eventually lead to the translocation of NF-κB complexes into the nucleus to regulate gene expression. The termination of NF-κB transcription activation in the nucleus occurs through negative feedback mechanisms by which resynthesized IκBα induced by NF-κB-mediated transactivation promotes NF-κB-IκBα complex formation, and shuttles NF-κB to the cytosol in its inactive form.

Importantly, there is a second level of regulation of NF-κB/Rel proteins mediated by post-translational modification, such as ubiquitination, prolyl isomerization, phosphorylation, and acetylation of NF-κB subunits 21, 22. This regulation controls the duration and amplitude of NF-κB activation through regulation of nuclear entry, DNA binding, transactivation, and the interaction of NF-κB proteins with other transactivators.

In the present study, we investigate molecular mechanisms of NF-κB transcriptional activation in the control of inflammatory cytokine expression by SPTBN1 in human HCC cells and the clinical implications of suppression of SPTBN1 and induction of inflammatory cytokine expression in predicting relapse-free survival of HCC patients. Our work reveals that SPTBN1 functions to control the stability of the p65 subunit of the NF-κB superfamily through collaboration of the suppressor of cytokine signaling 1 (SOCS1) protein, an E3 ligase of p65, and that this novel regulatory cascade might be an important molecular mechanism underlying the loss of tumor suppressive function of SPTBN1 during cancer development and progression.

## Materials and Methods

### Cell culture and transfection

All cells were grown in a humidifier in the presence of 5% CO_2_ at 37 °C and cell culture medium was supplemented with 10% fetal bovine serum, 50 units/mL of penicillin, 50 units/mL of streptomycin, and 4 mM of L-glutamine. Human HCC cell lines, PLC/PRF/5 (CRL-8024), and SNU-449 (CRL-2234), were purchased from American Type Culture Collection (ATCC) (Manassas, VA). siRNAs target SPTBN1 and control siRNA were transfected respectively into cells using Lipofectamine 3000 Transfection Reagent (ThermoFisher Scientific).The siRNA sequences are as follows: siRNA1 (SPTBN1-1), 5'-GGAAUUGCAGAGGACGUCUAGUAUC-3' (sense), 5'-GAUACUAGACGUCCUCUGCAAUUCC-3' (antisense); siRNA2 (SPTBN1-2), 5'-ACCUUCGAGAUGGACGGAUGCUCAU-3' (sense), 5'-AUGAGCAUCCGUCCAUCUCGAAGGU-3' (antisense); sictrl (control siRNA), 5'-GGAACGUGGAGUGCAGAUCUUAAUC-3' (sense), 5'-GAUUAAGAUCUGCACUCCACGUUCC-3' (antisense).

### RNA extraction and quantitative real-time reverse-transcription polymerase chain reaction (QRT-PCR)

Total RNA was isolated using RNeasy Mini Kit (Qiagen, Valencia, CA). cDNAs were synthesized using the TaqMan reverse transcription reagents (Applied Biosystems, Foster City, CA). For QRT-PCR analysis, 50 ng of cDNA was used as a template in triplicate reactions for each primer pair and the assay was performed using an iCycler iQ™ Real-Time PCR Detection System (Bio-Rad, Hercules, CA) with the primers as follows: Human IL-1β, 5'-GCTGAGGAAGATGCTGGTTC-3' (forward), 5'-TCCATATCCTGTCCCTGGAG-3' (reverse); human IL-1α, 5'-GTAAGCTATGGCCCACTCCA-3' (forward), 5'-AGGTGCTGACCTAGGCTTGA-3' (reverse); human IL-6, 5'-CTTCCAATCTGGATTCAATG-3' (forward), 5'-GTCAGGGGTGGTTATTGCAT-3' (reverse); human SPTBN1, 5'-ATCTAACGCACACTACAACCTG -3' (forward), 5'-TCAAGCACCTTTCCAATTCGT -3' (reverse); human p65, 5'-CCCACGAGCTTGTAGGAAAGG -3' (forward), 5'-GGATTCCCAGGTTCTGGAAAC-3' (reverse); human *GAPDH*, 5'-ACAGTCAGCCGCATCTTCTT-3' (forward) and 5'-GACAAGCTTCCCGTTCTCAG-3' (reverse); mouse IL-1β, 5'-GCAGCTATGGCAACTGTT-3' (forward), 5'-GAGCCTGTAGTGCAGTTGTC-3' (reverse); mouse IL-1α, 5'-TCGGGAGGAGACGACTCTAA-3' (forward), 5'-GTGCACCCGACTTTGTTCTT-3' (reverse); mouse IL-6, 5'-GCAAGAGACTTCCATCCAGT-3' (forward), 5'-CATGTACTCCAGGTAGCTAT-3' (reverse); and mouse GAPDH, 5'-TGTTCCTACCCCCAATGTGT-3' (forward), 5'-CCCTGTTGCTGTAGCCGTAT-3' (reverse).

### Immunoprecipitation and Western blotting

Western blot analysis was performed as described previously 8. Primary antibodies used for Western blot analysis are: SPTBN1; V5 epitope (R960-25) (ThermoFisher Scientific); p65 (8242, D14E12), IκBα (4814, L35A5), phosphor-IκBα (9246, 5A5) (Cell Signaling Technology); SOCS1 (sc-9021, H-93; sc-518028, E-9), Lamin B (sc-6217), α-Tubulin, HA-probe (sc-805, Y-11) (Santa Cruz Biotechnology); COMMD1, PIN1, PPARγ (Proteintech Group, Inc.); actin (A5316) (Millipore Sigma) and Ubiquitin (13-1600) (ThermoFisher Scientific). Secondary antibodies used are peroxidase-conjugated anti-mouse or anti-rabbit IgG (GE Healthcare Bio-Sciences).

For immunoprecipitation, cell supernatants were incubated with antibodies at 4 °C overnight. The immune complexes were recovered with protein G-Sepharose beads, which were then washed four times with lysis buffer; the immunoprecipitates were then subjected to Western blot analysis. Antibodies used for immunoprecipitation are mouse IgG, rabbit IgG (Sigma-Aldrich); SPTBN1; SOCS1 (ab9870) (Abcam); V5 epitope (R960-25) (ThermoFisher Scientific); IκBα (ThermoFisher Scientific) and p65 (sc-8008, F-6) (Santa Cruz Biotechnology).

### Fluorescence activated cell sorting (FACS) analysis

Single cell suspensions of liver, spleen, bone marrow and peripheral blood samples were prepared from wild type (WT) and *Sptbn1^+/-^* mice. Mice were euthanatized with CO_2_ and their liver, spleen, bone marrow, and peripheral blood were collected. Livers and spleens were minced with scissors and a razor knife and then filtered with 40 μm nylon mesh strainer (BD Biosciences, San Jose, CA). All single suspended cells were depleted of red blood cells using 1×RBC lysis buffer for 5 minutes on ice. Single cells were suspended in Dulbecco's PBS (D-PBS) (Mediatech, Inc.) and stained with fluorescence-conjugated antibodies: PE-Cy7-CD45, APC-CD4, APC-F4/80, Alexa Fluor® 488-Ly-6G/Ly-6C (Gr-1), Brilliant Violet 421-CD11b, Brilliant Violet 421-CD25, and Alexa Fluor® 488-FOXP3 (Biolegend). Subsequent to being washed, cells were analyzed using a BD LSRII flow cytometer (BD Biosciences, San Jose, CA). Profiles of lymphoid and myeloid cells in CD45-gated leukocytes were assessed using FlowJo7 software (FlowJo, Williamson Way, Ashland, OR).

### Primary culture of single cell suspension of whole liver tissues from mice

Age (6 to 8 months)- and gender-matched WT and *Sptbn1^+/-^* mice were euthanized with CO_2_, and mice liver tissues were collected and placed in a cell culture dish in 1 mL of calcium- and magnesium-free Hank's Balanced Salt Solution (HBSS), supplemented with 25 mM Hepes, 0.2 mM EDTA, 50 units/mL of penicillin, and 50 units/mL of streptomycin. Livers were then minced with scissors and a razor knife and collected into 50 mL centrifuge tubes filled with 50 mL HBSS. Minced liver pieces were washed with HBSS several times until the HBSS solution became transparent. The liver pieces were then collected and digested with 0.1% type I and type IV collagenase solution at 37 °C for 50 minutes. The cell suspensions were collected, centrifuged, and resuspended in Dulbecco's Modified Eagle Medium (DMEM) supplemented with 10% fetal bovine serum, glutamine (4 mM), 50 units/mL of penicillin, and 50 units/mL of streptomycin. The single cell suspension of liver tissues at a density of 5 × 10^5^ cells was inoculated into 25 cm^2^ cell culture flasks coated with 0.1% type I collagen (Sigma-Aldrich, St. Louis, MO). All cells were cultured at 37 °C in a water-saturated atmosphere of 5% CO_2_ in air. The supernatants were then collected 48 h after inoculation and subjected to ELISA analysis.

### Measurement of cytokines by enzyme linked immunosorbent assay (ELISA)

Concentrations of IL-1α, IL-1β and IL-6 in cell culture supernatants generated from cultivated human HCC cells, or from the primary culture of single cell suspensions of whole liver tissues from mice, were measured using commercially available ELISA kits (Biolegend, San Diego, CA and eBioscience, Inc., San Diego, CA). Cytokine release was indicated in unit of pg/mL following triplicate analysis of each sample.

### Analysis of gene array database from human HCC

We performed correlation analysis of SPTBN1, IL-1α*,* IL-1β and IL-6 gene expression using three public human gene array databases from Gene Expression Omnibus (GEO). The human hepatitis viruses B (HBV) data set considered for this analysis was GSE14520 obtained from GEO 23, 24. The human hepatitis viruses C (HCV) data set considered was GSE6764 25. The raw data set of gene expression profiling from this study was processed and uploaded to a bioinformatics platform, G-DOC Plus (Georgetown Database of Cancer), along with public clinical data from the study 26. After initial exploratory analysis on G-DOC Plus, we performed correlation analysis using Pearson correlation tests with statistical analysis tool, R 27. The alcohol-induced HCC dataset was GSE62232, which was found in a publicly available alcohol induced HCC dataset in NCBI's repository GEO. The dataset contained a total of 81 HCC and 10 non tumor liver samples. Out of these, 22 patients had an etiology of alcohol alone were considered for analysis. After background correction and summarization, RMA normalization was applied and the data was converted into the log2 scale. The gene expression data for SPTBN1, IL-1α*,* IL-1β and IL-6 were extracted for the 22 alcohol- induced HCC patients. A Pearson correlation test was applied and scatter plots were done. All analysis was performed using the R statistical framework.

We further studied the clinical relevance of *SPTBN1*, *IL-1α, IL-1β* and *IL-6* expression with relapse-free survival using the human HBV-induced HCC gene array database GSE14520 (the HCV-induced HCC data set did not have survival data, and hence could not be used for this analysis) (see Supplementary Materials) 23, 24, 28.

### Luciferase assay

Plasmid 3×-κB-L (addgene, Cambridge MA), which comprises a fragment of minimal fos promoter element and three copies of the major histocompatibility complex (MHC) class I κB element TGGGGATTCCCCA, was generated by ligating the fragment to pGL2-Basic vector 29. The promoter activity of 3×-κB-L constructs was measured by a Dual-Luciferase Reporter (DLR) Assay System (Promega, Madison, WI).

### Electrophoretic mobility shift assay (EMSA)

Nuclear proteins from PLC/PRF/5 cells were incubated with biotinylated oligonucleotide at room temperature for 20 min. Complexes were then separated on a 6% acrylamide none-denaturing gel and transferred to a nylon membrane for detection using the light-shift electrophoretic mobility shift assay (EMSA) kit (Thermo Fisher Scientific Inc.). The oligonucleotide probe sequence to detect NF-κB binding was 5'-GATCCATTAGGGGATGCCCCTCAT-3' 30.

### Chromatin immunoprecipitation quantitative real time PCR (ChIP-qPCR)

In brief, LV-SPTBN1-sh stable transfected PLC/PRF/5 cells and its control cells were seeded into a 150 mm culture dish at 90% confluence, and one day later, they were cross-linked with 1% formaldehyde for 15 min at room temperature, followed by genomic DNA fragmentation using a sonication apparatus. Then, the chromatin fragments were immunoprecipitated with 5 μg of an antibody against SMAD2/3 (8685S, CST, Danvers, MA, USA), an antibody against IgG (3900S, CST, Danvers, MA, USA) or antibody against H3 (4620S, CST, Danvers, MA, USA). DNA extraction was performed using a Qiagen Purification kit. Real-time PCR analysis was performed with primers amplifying the promoters of IL-6, 5'-TCTGCAAGATGCCACAAGGT-3' (forward), 5'-TGAAGCCCACTTGGTTCAGG-3' (reverse); and IL-1β: 5'-CTTCCACTTTGTCCCACAT-3' (forward), 5'-GTGCAGTTGATGTCCACAT-3' (reverse).

### Construction of mouse models of hepatocellular carcinoma

The evidences demonstrated that 3,5-diethoxycarbonyl-1,4-dihydrocollidine (DDC) induced liver inflammation causes increased expression of pro-fibrogenic and pro-inflammatory cytokines and activation of Kupffer cells, possibly even leading to HCC according to the specific gene mutation 31. To study whether *Sptbn1^+/-^* mice are susceptible to the DDC induced malignancy in liver, six to eight weeks old WT (n = 7) and *Sptbn1^+/-^* (n = 14) mice were fed with a diet containing 0.1% DDC for 2 months. In addition to the continued treatment with 0.1% DDC-containing diet, 7 of 14 *Sptbn1^+/-^* mice were randomly selected to receive intraperitoneal injection with JSH-23 (2 mg/kg, p65 inhibitor), and the other mice were injected with vehicle as control. 1 month later, mice were sacrificed for observing liver tumor incidence and liver tissue specimens were harvested.

### Histological staining

Immunohistochemical staining of liver tissues from mouse (6 to 8 months old or >15 months), and patients with HCC was performed to detect p65, Foxp3, F4/80 and SPTBN1 signal. Five-micron sections from formalin fixed paraffin embedded tissues were de-paraffinized with xylenes and rehydrated through a graded alcohol series. Heat induced epitope retrieval (HIER) was performed by immersing the tissue sections at 98 °C for 20 minutes in 10 mM citrate buffer (pH 6.0), with 0.05% Tween.

Immunohistochemical staining was performed using the Histostain Plus Kit from Zymed/Invitrogen according to manufacturer's instructions. Briefly, slides were treated with 3% hydrogen peroxide for 10 minutes. Endogenous biotin was blocked using an avidin/biotin blocking kit from Invitrogen. Each slide was then treated with Zymed solution A for 10 minutes, exposed to primary antibodies for p65 (1:200, Santa Cruz Biotechnology, Inc., Dallas, TX), Foxp3 (1:200, Abcam, Cambridge, MA), F4/80 (1:200, Proteintech Group, Inc. Rosemont, IL) and SPTBN1 (1:300, Life Technologies, Grand Island, NY)), respectively for 1 hour at room temperature, treated with Zymed solutions B and C for 10 minutes, and finally exposed to DAB Chromagen (Dako) for 5 minutes. Slides were counterstained with Hematoxylin (Fisher, Harris Modified Hematoxylin) at a 1:17 dilution for 2 min at room temperature, exposed to bluing solution in 1% ammonium hydroxide for 1 min at room temperature, dehydrated, and mounted with AcryMount. Consecutive sections with the omitted primary antibody served as negative controls.

Haematoxylin and eosin (HE) staining and Gomori's reticular staining were performed to evaluate the carcinogenesis of liver tissues. Gomori's reticular staining was performed to identify reticular fibers.

### Analysis of The Cancer Genome Atlas (TCGA) database and the effect of SPTBN1, SOCS1 and p65 on HCC prognosis

Clinicopathological data were available from TCGA database. The correlation analysis of SPTBN1 and SOCS1 was analyzed by Gene Expression Profiling Interactive Analysis (GEPIA, website: http://gepia.cancer-pku.cn/detail.php?clicktag=correlation) using hepatocellular carcinoma retrieved form TCGA data. Recurrence-free survival (RFS, n = 313) and overall survival (OS, n = 364) were determined using the Kaplan-Meier method (website: http://kmplot.com/analysis/index.php?p=service&cancer=liver_rnaseq) by retrieving RNA-seq data of hepatocellular carcinoma provided by Menyhart O et al 32.

### Animal care

All studies involving the use of mice were performed under a protocol (12-032) approved by the Georgetown University Institutional Animal Care and Use Committee (IACUC). Animals received humane care according to the criteria outlined in the Guide for the Care and Use of Laboratory Animals. *Sptbn1^+/-^* mice were developed in Dr. Lopa Mishra's laboratory 4.

### Statistical analysis

Student's *t* test was performed on QRT-PCR, ELISA, Luciferase assay and Western blot quantification analyses. Differences were regarded as statistically significant if *P* < 0.05 (*), and as highly statistically significant if *P* < 0.01 (**) or *P* < 0.001 (***). The statistical analyses performed on the gene array databases were Pearson correlation tests and Cox proportional hazards model; these were performed in the statistical computing tool R 27, 28.

Other methods are described in the Supplementary Materials.

## Results

### Loss of SPTBN1 induces inflammatory cytokine production, and SPTBN1 is inversely correlated with the expression of IL-1α, IL-1β and IL-6 in HCV- and HBV- as well as alcohol-induced HCC

**Figure 1:**
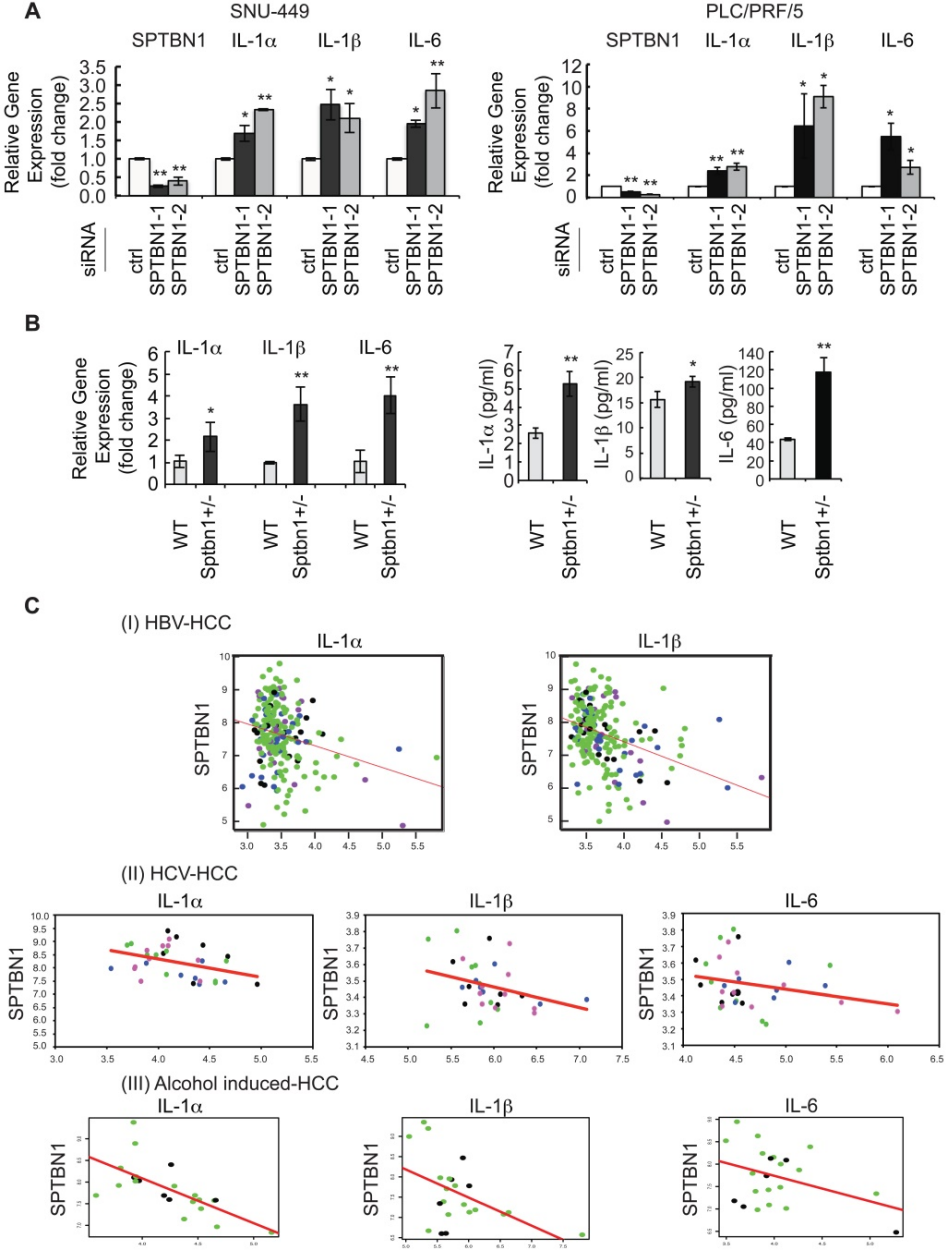
An increase in inflammatory cytokines was observed in human HCC cells and in liver tissues of *Sptbn1^+/-^* mice, and an inverse correlation of *SPTBN1* with *IL-1α, IL-1β* and *IL-6* was observed in human HCV- and HBV- induced HCC as well as alcohol- induced HCC tissues. (A) PLC/PRF/5 and SNU449 cells transiently transfected with control siRNA or siRNA to SPTBN1 were cultured for 48 hours and subjected to QRT-PCR analysis for IL-1α, IL-1β and IL-6 mRNA. Significance of the mean value difference was determined using a Student's *t* test (*P < 0.05; **P < 0.01 compared with the control siRNA group). (B) QRT-PCR analysis of IL-1α, IL-1β and IL-6 mRNA in liver tissues from WT and *Sptbn1^ +/-^* mice (n = 5) (left). The normalized fold-change (mean ± S.D.) (comparing to WT group) were shown. The release of cytokines in cell culture medium of primary culture from single cell suspensions prepared from mouse liver tissues was measured by ELISA assay (right). The significance of the difference between WT and *Sptbn1^+/-^* livers with respect to cytokines in mRNA levels in tissues and the released protein expression in culture was determined using a Student's *t* test (*P < 0.05; **P < 0.01). (C) Correlation analysis of gene expression in human HBV- and HCV- induced HCC tissues using G-DOC platform. Tumor stages of the analyzed human HBV- and HCV-induced HCC cases are indicated by different dot colors: blue, very early HCC; green, early HCC; purple, very advanced HCC; and black, advance HCC. The human HBV-induced HCC data set consisted of 225 liver tumor samples and 220 paired non-tumor samples with clinical and gene expression data (I). The human HCV-induced HCC data set consisted of 75 samples representing stepwise carcinogenic processes from pre-neoplastic lesions to HCC. Of these, 33 samples including very early, early, advanced, and very advanced HCC were considered for analysis (II). The human alcohol-induced HCC data set consisted of 22 samples. Raw gene expression data was obtained from Gene expression omnibus (GEO). The correlation scatter plots were colored data based “Edmonson Grade” clinical attribute - which had values either Grade I/II-black dots or Grade II/III-green dots (III). Correlation coefficients (r) and p-values between two groups of genes selected from *SPTBN1*,* IL-1α, IL-1β* and* IL-6* were obtained using Pearson correlation tests.

During the course of exploring the molecular mechanisms of SPTBN1 loss in the liver cancer formation, we performed microarray analysis in SNU-449 HCC cell line treated with SPTBN1 siRNA. Among the differential expressed inflammatory genes as shown in the heat map, we observed that transcription levels of the IL-6 gene increased significantly compared with cells following control siRNA treatment (Supplementary Figure S1A). IL-6 is a pleiotropic cytokine implicated in inflammation-associated diseases 11, 33. This observation prompted us to analyze the expression of related inflammatory cytokines including IL-1α, IL-1β, and IL-6 in HCC cell lines following inhibition of SPTBN1. Upon transient transfection of siRNA targeting SPTBN1 in HCC cell lines, compared with cell lines treated with control siRNA, QRT-PCR analysis showed that IL-1α and IL-1β in addition to IL-6 mRNA levels were significantly increased 2-6 fold in PLC/PRF/5 (*P* < 0.01 for IL-1α gene; *P* < 0.05 for IL-1β and IL-6 genes) and 2-4 fold in SNU-449 cells (*P* < 0.01 for IL-1α and IL-6 genes; *P* < 0.05 for IL-1β gene) (Figure 1A). The 2-4 fold increase in IL-1α, IL-1β and IL-6 mRNA levels, as well as the release of these cytokines into primary cultures of single cell suspensions from mouse liver tissues were further observed in *Sptbn1^+/-^* mice compared to those derived from WT mice (*P* < 0.05 for IL-1β gene; *P* < 0.01 for IL-1α and IL-6 genes) (Figure 1B). To further examine whether the increased inflammatory cytokines upon suppression of SPTBN1 is through the regulation of TGF-β signaling pathway, we carried out ChIP-qPCR analysis by using PLC/PRF/5 cells with SPTBN1 silencing. Our results demonstrated that binding of SMAD2/3 to the promoter of IL-6 or IL-1β gene was not affected after SPTBN1 knockdown, indicating that SPTBN1 mediated suppression of IL-6 and IL-1β genes were not through SMAD2/3-mediated TGF-β signaling pathway (Figure S1B).

Next, we assessed whether the gene expressions of IL-1α*,* IL-1β and IL-6 were inversely correlated with SPTBN1 gene expression in HCV- and HBV- as well as alcohol-induced HCC tissue samples. Correlation analysis showed a significant negative correlation between *SPTBN1* and *IL-1α* (correlation coefficient = -0.245, p-value = 2.37E-4); and between *SPTBN1* and *IL-1β* (correlation coefficient = -0.355, p-value = 6.56E-08) in the HBV-induced HCC study (Figure 1C-I). Results also showed significant negative correlation between *SPTBN1* and *IL-1α* (correlation coefficient = -0.386, p-value = 0.026) in the HCV-induced HCC study. Although the correlation of *SPTBN1* with *IL-6* (correlation coefficient = -0.258, p-value = 0.147), and *SPTBN1* and *IL-1β* (correlation coefficient = -0.307, p-value = 0.082) did not reach statistical significance in HCV-induced HCC, the trend of inverse relationship is clearly seen in HCV-induced HCC (Figure 1C-II). In alcohol-induced HCC,* IL-1α* and *IL-1β* are significantly negatively correlated with *SPTBN1* (*IL-1α vs SPTBN1*: correlation coefficient = -0.67 and p-value = 0.001; *IL-1β vs SPTBN1*: correlation coefficient = -0.49 and p-value = 0.001) but the negative correlation of *IL-6* with *SPTBN1* did not reach statistical significance (correlation coefficient = -0.39 and p-value = 0.077) (Figure 1C-III). These correlations were independent of human HCC stage, suggesting that the downregulation of *SPTBN1* and the upregulation of *IL-1α*, *IL-1β* and *IL-6* occur in HCC throughout all stages of cancer progression, and also irrespective of human hepatitis virus status (Figure 1C).

We further studied the clinical relevance of *SPTBN1*, *IL-1β* and *IL-6* expression to relapse-free survival using the human HBV-induced HCC gene array database. We found that among 212 tumor samples in the GSE14520 (HBV-induced HCC) study, 72 HCC samples had the expression of *SPTBN1* below the median level among the whole group of samples and expression of *IL-1β* above the median level among the whole group (*SPTBN1* low/*IL-1β* high); 41 HCC samples had the expression of SPTBN1 low/IL-6 high among the whole group; 63 HCC samples had the expression of SPTBN1 low/IL-1α high among the whole group. Of 212 HCC samples, 36 (17%) had all three cytokines (IL-1β, IL-6, and IL-1α) above the median level and SPTBN1 below the median level, while 45-54% had two of the three cytokines above the median level and SPTBN1 below the median level. The Cox proportional hazards model showed that in the cohort of HCC patients with *SPTBN1* low/*IL-1β* high, the ratio of *SPTBN1* over *IL-1β* correlates with relapse free survival with a hazard ratio of 0.634 indicating the loss of *SPTBN1* and/or increased level of *IL-1β* correlates with shorter relapse free survival (p = 0.03916). The model also showed that in the cohort of HCC patients with *SPTBN1* low/*IL-6* high, the ratio of *SPTBN1* over *IL-6* correlates with relapse free survival with a hazard ratio of 0, indicating the loss of *SPTBN1* and/or increased level of *IL-6* correlates with shorter relapse free survival (p = 0.04901) (Supplementary Table S1-4).

### Loss of SPTBN1 increases the protein level, nuclear retention, and function of the p65 subunit of NF-κB in human HCC cells and mouse liver

**Figure 2:**
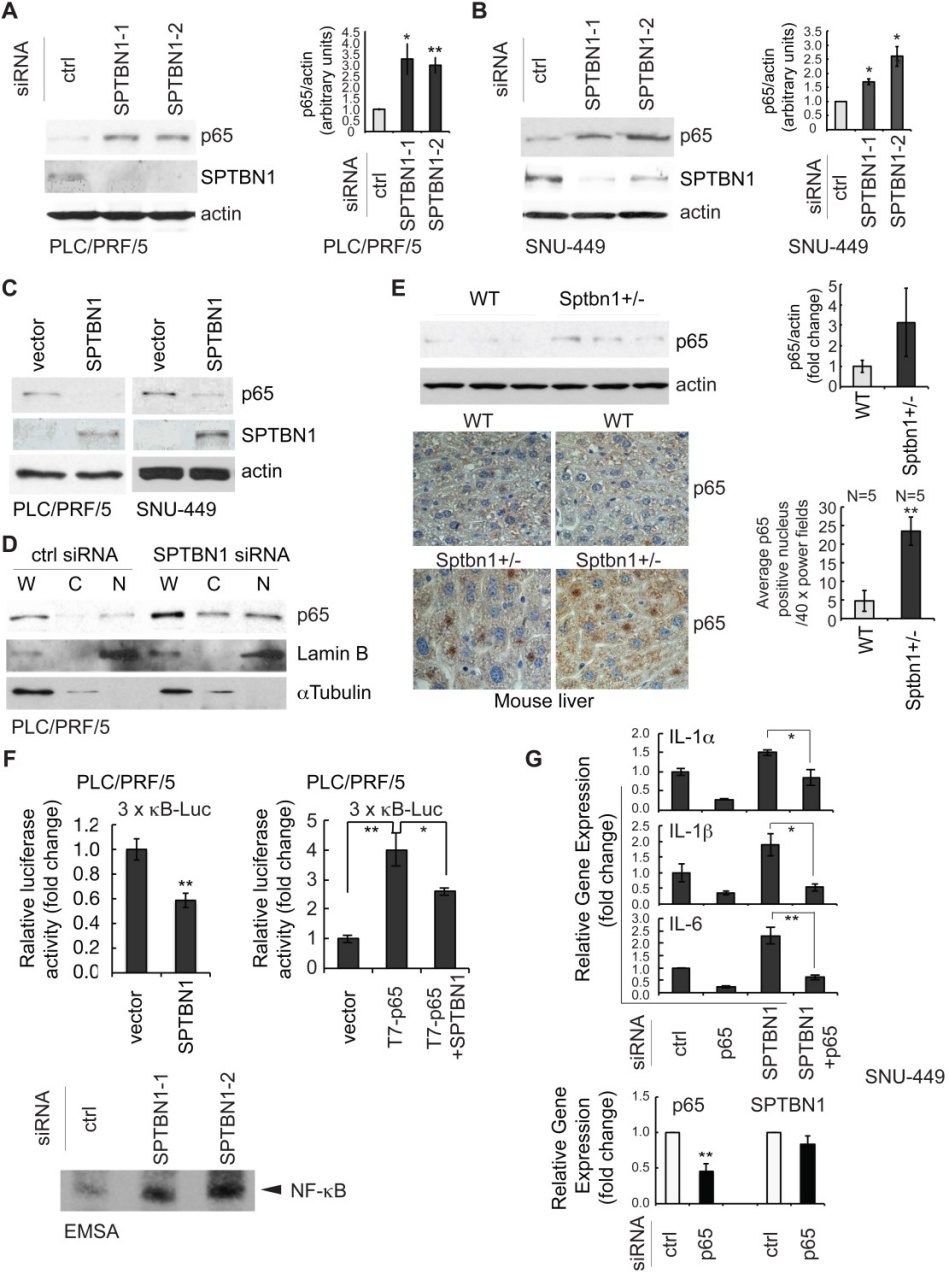
The suppression of inflammatory cytokines by SPTBN1 was mediated through inhibition of p65. (A and B) PLC/PRF/5 (A) and SNU-449 (B) cells were transiently transfected with siRNAs for 48 h and were then subjected to Western blot analysis. Data is representative of three independent experiments. The intensities of p65 and actin in two to three independent Western blotting were measured by ImageJ software, and the ratios of p65/actin were analyzed. Significance of the difference was evaluated using the Student's *t* test (*P < 0.05; **P < 0.01). (C) SNU-449 and PLC/PRF/5 cells were transiently transfected with empty vector or SPTBN1 plasmid for 48 h and were analyzed by Western blotting. (D) PLC/PRF/5 cells were transfected with control siRNA or siRNA to SPTBN1 for 48 hours and the cells were fractionated into cytoplasmic [C] and nuclear [N] fractions and subcellular distribution of p65 was assessed by Western blot analysis. The purity of nuclear fractions was verified with antibodies to Lamin B and αTubulin, respectively. W: whole cell lysate; C: cytoplasmic fraction; N: neuclear fraction. (E) Liver tissues from WT and *Sptbn1^+/-^* mice were analyzed by Western blotting (upper left) and immunohistochemistry staining (lower left) by antibodies as indicated. Quantification of the average p65 positive nuclei in 40 × power fields were shown (mean ± S.D.) (right). (F-upper) PLC/PRF/5 cells were transfected with empty vector or SPTBN1 (left), or were transfected with empty vector, T7-p65 plasmid without or with SPTBN1 plasmid (right), together with 3×κB-L reporter plasmid and pRL-TK for 48 h and were lysed to measure luciferase activity. Normalized (to the empty vector group) relative luciferase activity is shown (mean ± S.D.). Significance of the difference was evaluated using the Student's *t* test (*P < 0.05; **P < 0.01). (F-lower) DNA binding activity of endogenous NF-κB in HCC cells. Neuclear extracts of PLC/PRF/5 cells which were transiently transfected with siRNAs as indicated were subjected to EMSA analysis by using biotin-labeled DNA probe corresponding to NF-κB consensus DNA sequence as described in Methods. (G) SNU-449 cells were transiently transfected with siRNAs as indicated for 48 h. Cells were then analyzed by QRT-PCR for mRNA levels of IL-1α, IL-1β, IL-6 (upper panels) and p65 as well as SPTBN1 (lower panel).

We next explored the possible regulation of NF-κB, the upstream regulator of IL-1*α*, IL-1β, and IL-6, by SPTBN1. We examined the expression of p65, one of the important subunits of NF-κB in HCC cells and found that transient transfection of SNU-449 and PLC/PRF/5 cells with SPTBN1 siRNA augmented the cellular expression level of p65 protein compared with cells transfected with control siRNA (Figure 2A-B). Furthermore, overexpression of SPTBN1 in SNU-449 and PLC/PRF/5 cells inhibited the expression of p65 (Figure 2C). Further analysis indicated that the protein level of p65 in the cell nucleus was increased in PLC/PRF/5 upon the suppression of SPTBN1 in contrast with cells treated with control siRNA, as shown in the analysis of p65 subcellular distribution and co-immunofluorescence staining (Figure 2D and Figure S2). We also observed that the protein level of p65 was elevated in *Sptbn1^+/-^* mouse livers compared with that of age-matched WT mouse livers (Figure 2E, upper panels). Immunohistochemical analysis indicated that heterozygous loss of SPTBN1 is associated with the nuclear accumulation of p65 as assessed by the significantly increased numbers of cells with p65-positive nuclear staining by five-fold (*P* < 0.01) in livers of *Sptbn1^+/-^* mice compare to WT mice liver (Figure 2E, lower panels).

Next, we examined the transcription activity of NF-κB by measuring luciferase reporter activity of the 3×-κB-L construct, which was shown by Mitchell et al, 1995, to be responsive to NF-κB transcription factors 29. Overexpression of SPTBN1 led to significant inhibition of NF-κB-dependent luciferase reporter activity of 3×-κB-L in PLC/PRF/5 cells with and without exogenous expression of p65 [Figure 2F (upper left), *P* < 0.01, and Figure 2F (upper right), *P* < 0.05, respectively]. We also found that the DNA binding activity of NF-κB was highly increased in PLC/PRF/5 cells following knockdown of SPTBN1 as determined by electrophoretic mobility shift assay (EMSA) analysis using NF-κB consensus DNA sequence 30 (Figure 2F lower).

We further questioned whether the increased expression of p65 protein is involved in the induction of cytokines IL-1α, IL-1β and IL-6 that was observed in HCC cells and in liver tissues from *Sptbn1^+/-^* mice following suppression or impairment of SPTBN1. The mRNA levels of IL-1α, IL-1β and IL-6 in SNU-449 (Figure 2G, upper) and PLC/PRF/5 cells (Figure S3A) as well as the protein release of IL-1α and IL-6 in SNU-449 cells (Figure S3B) were no longer induced after treatment with siRNAs targeting both SPTBN1 and p65. Suppression of p65 by siRNA specific to p65 in SNU-449 cells showed no effect on the expression of SPTBN1 (Figure 2G, lower). These results indicate that p65, activated in HCC cells and mouse liver tissues upon the suppression of SPTBN1, is required for the increased production of inflammatory cytokines that follows SPTBN1 suppression.

### SPTBN1 induces ubiquitination and degradation of p65 through the regulatory effects of SOCS1

**Figure 3:**
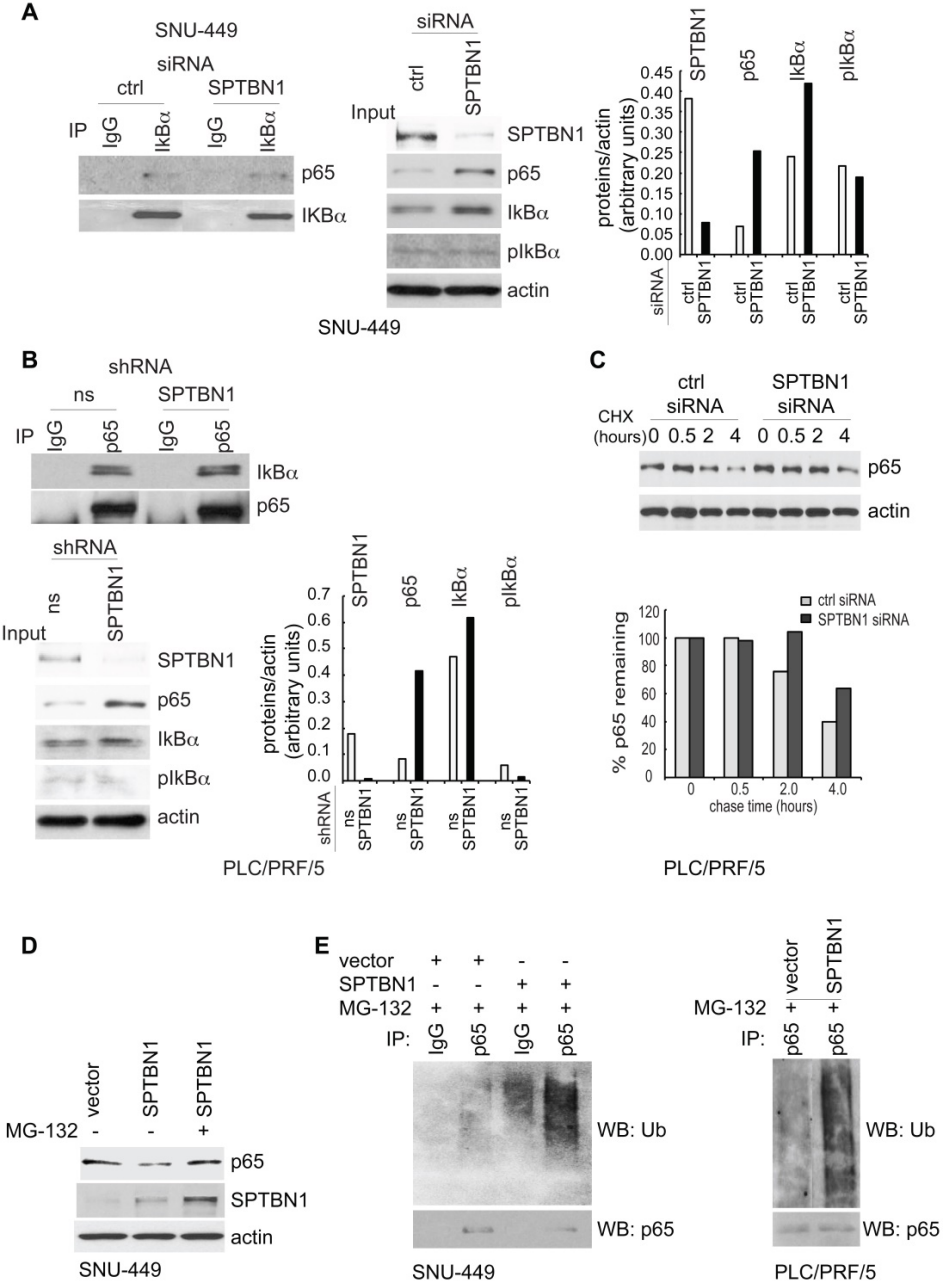
SPTBN1 induces ubiquitination and degradation of p65. (A and B) Binding of p65 with IκBa kept identical in HCC cells upon the inhibition of SPTBN1. SNU-449 cells were transiently transfected with siRNAs as indicated for 48 h (A) and PLC/PRF/5 cells stably transduced with SPTBN1 shRNA or non-specific (ns) shRNA were cultured for 48h (B). Cells were then subjected to immunoprecipitation (IP) and immunoblotting with antibodies as indicated. Whole cell lysates of above cells (inputs) were analyzed by Western blotting by antibodies as indicated. Data are representative of two to three independent experiments with similar results. The intensities of SPTBN1, p65, IκBα, pIκBα and actin were measured by ImageJ software, and the relative expression level of each protein/actin was generated. (C) PLC/PRF/5 cells were transiently transfected as indicated for 48h. Cells were then treated with cycloheximide (CHX) for the indicated time periods and were analyzed for the stability of p65 by Western blotting (upper). The intensities of p65 and actin were measured by ImageJ software, and the relative expression units of p65/actin were generated and normalized relative to the expression unit in 0 h. The percentage remaining of p65 expression (compare to zero hour) was then calculated (lower). (D) SNU-449 cells were transfected as indicated for 48 h. Cells were then treated without or with 10 µM MG132 for 4 h before cell lysis and analyzed by Western blotting. (E) SNU-449 cells and PLC/PRF/5 cells were transiently transfected as indicated for 48 h. Cells were then treated with 10µM MG132 for 4 h before cell lysis. Cells were immunoprecipitated with IgG or antibody to p65 and the immunoprecipitates were then immunoblotted with anti-ubiquitin antibody to detect ubiquitination of endogenous p65.

The above results prompted us to study the mechanism by which SPTBN1 regulates p65. First, we investigated whether the increased nuclear expression of p65 was a result of dissociation of IκBα from p65 following phosphorylation of IκBα. Thus, we examined the amount of p65 that was bound to IκBα and found that the binding amount was the same whether SNU449 cells were transfected with control siRNA or with siRNA to SPTBN1 (Figure 3A, left). Similarly, the amount of IκBα associated with p65 was identical in PLC/PRF/5 cells stably transduced with either SPTBN1 shRNA or ns shRNA (Figure 3B, upper). Further analysis revealed that the level of phosphorylated IκBα was not affected in SNU-449 cells treated with SPTBN1 siRNA compared with cells transfected with control siRNA, or in PLC/PRF/5 cells stably transduced with SPTBN1 shRNA. Instead, we observed an increase in IκBα protein upon the inhibition of SPTBN1 (Figure 3A, middle and right and Figure 3B, lower). Next, we examined p65 gene expression, and found that p65 mRNA levels slightly decreased in both HCC cells and mouse liver tissues following the knockdown or impairment of SPTBN1 (Figure S4A). These results indicate that the increased protein level of p65 was neither induced by the phosphorylation and degradation of its upstream inhibitor IκBα nor the transcriptional regulation of the p65 gene, but may instead be the result of post-translational modification of p65 protein, which occurred when SPTBN1 was inhibited 22.

We thus investigated if SPTBN1 regulates the ubiquitination and degradation of p65. First, we examined if the accumulated p65 protein is associated with increased stability of p65. We found that the decay rate of p65 was delayed for 2 h in cells with SPTBN1 knockdown compared with cells treated with control siRNA (Figure 3C). Overexpression of SPTBN1 in SNU-449 cells suppressed the expression of p65 protein, a loss which was prevented when the cells were treated with the proteasome inhibitor, MG132, indicating that SPTBN1 induces proteasome-mediated degradation of p65 (Figure 3D). Further analysis showed that overexpression of SPTBN1 caused significant ubiquitination of endogenous p65 compared with cells transfected with empty vector in SNU-449 cells and PLC/PRF/5 cells (Figure 3E). These results demonstrated that SPTBN1 inhibits p65 protein expression by inducing ubiquitination and proteasome-mediated degradation of p65.

**Figure 4:**
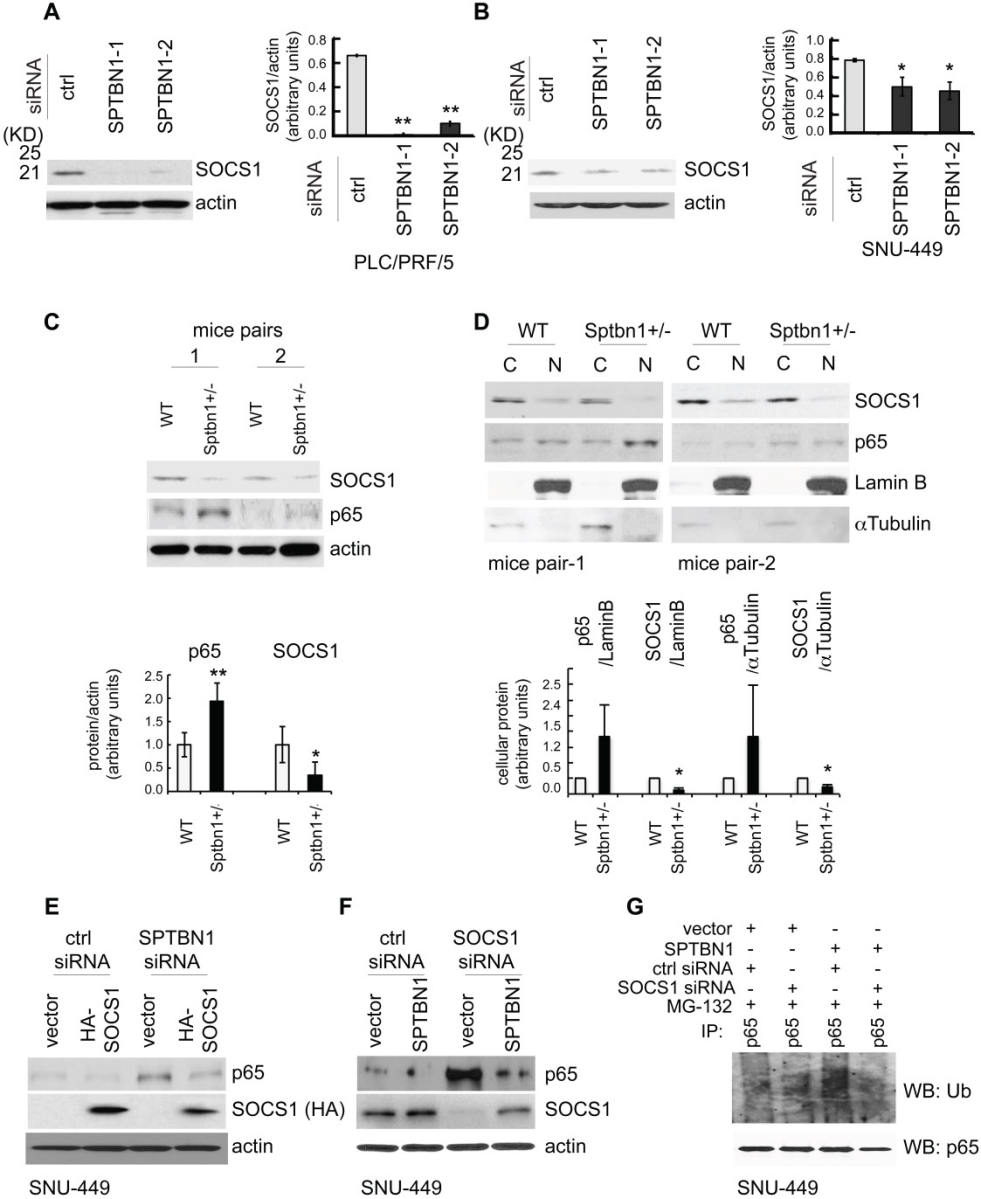
SPTBN1 enhances the expression of SOCS1, which was required for the regulation of p65 by SPTBN1. (A-B) PLC/PRF/5 (A) or SNU-449 (B) cells were transiently transfected with siRNAs as indicated. Cells were then subjected to Western blot analysis. The relative intensities of SOCS1 to actin in two to three independent Western blotting were analyzed as in Figure 2A and 2B. Significance of the difference was evaluated using the Student's *t* test (*P < 0.05; **P < 0.01). (C-D) Liver tissues from two pairs of age- and gender-matched WT and *Sptbn1^+/-^* mice were analyzed by Western blotting (C). Liver tissues from two pairs of age- and gender-matched WT and *Sptbn1^+/-^* mice were lyzed and fractionated into cytoplasmic [C] and nuclear [N] fractions, and subcellular distribution of p65 and SOCS1 in each fraction was assessed by Western blotting (D). The relative intensities of p65 and SOCS1 were analyzed as in Figure 2A and 2B. (E) SNU-449 cells were transiently transfected as indicated for 48 h and were then analyzed by Western blot analysis. (F) SNU-449 cells were transiently transfected with control siRNA or siRNA to SOCS1 together with empty vector or SPTBN1 plasmid for 48 h. Cells were then analyzed by Western blot analysis. (G) SNU-449 cells were transiently transfected with control siRNA or siRNA to SOCS1 together with empty vector or SPTBN1 plasmid for 48 h. Cells were then treated with 10µM MG132 for 4 h before cell lysis. Cells were immunoprecipitated with antibody to p65 and the immunoprecipitates were then immunoblotted with anti-ubiquitin antibody to detect ubiquitination of endogenous p65.

Next, we studied the mechanism by which SPTBN1 regulates the ubiquitination and proteasome-mediated degradation of p65. We questioned if SPTBN1 induces ubiquitination of p65 via regulation of the p65 ubiquitin ligase system. We examined several well-identified ubiquitin ligases of p65, including COMMD1, PIN1, PPARγ, and SOCS1 22, 34-37, and found that suppression of SPTBN1 led to downregulation of SOCS1 in PLC/PRF/5 cells (Figure 4A) and SNU-449 cells (Figure 4B and Figure S4B). We then analyzed the expression of SOCS1 in liver tissues from WT and *Sptbn1^+/-^* mice. Western blot analysis of two pairs of liver tissues from age- and gender-matched WT and *Sptbn1^+/-^* mice indicated that the level of SOCS1 protein decreased in liver tissues of *Sptbn1^+/-^* mice compared with age-matched WT mice (Figure 4C). We further analyzed the expression of p65 and SOCS1 in liver tissues after cytoplasmic and nuclear fractionation from two pairs of age- and gender-matched WT and *Sptbn1^+/-^* mice, and observed a clear inverse correlation between p65 and SOCS1. Thus, p65 was upregulated and SOCS1 was diminished in liver nuclear fractions from *Sptbn1^+/-^* mice compared with their WT counterparts (Figure 4D).

We next addressed whether SOCS1 is involved in the regulation of p65 by SPTBN1. Overexpression of SOCS1 markedly abolished the induction of p65 resulting from the suppression of SPTBN1 (Figure 4E). Conversely, inhibition of SOCS1 by siRNA silencing abolished the suppression of p65 as well as the ubiquitination of p65 following overexpression of SPTBN1 in SNU-449 cells (Figure 4F-G). These data clearly demonstrate that expression of SOCS1 is required for the inhibition of p65 by SPTBN1.

### SPTBN1 binds to SOCS1 and overexpression of SPTBN1 increases stability of SOCS1

**Figure 5:**
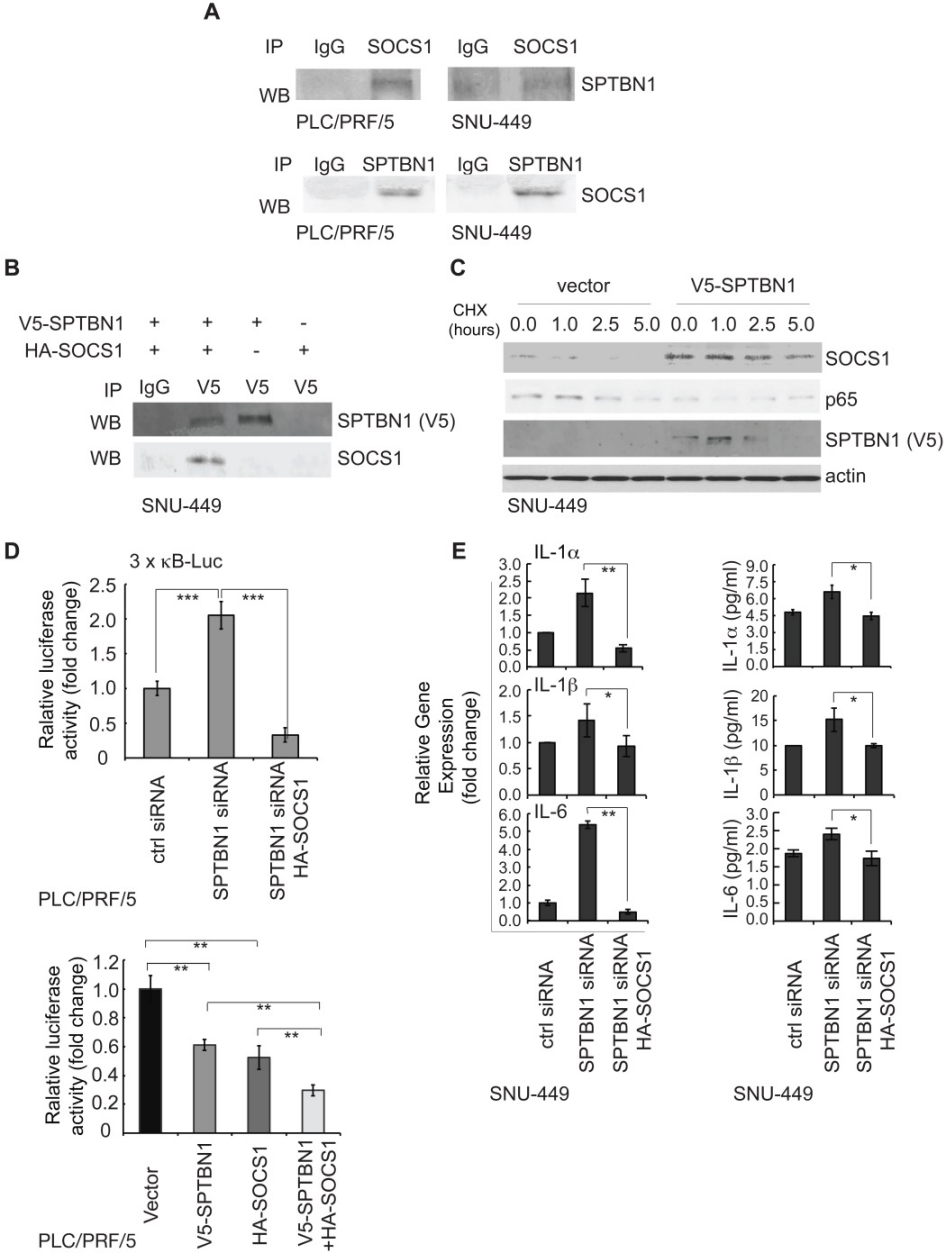
SPTBN1 binds to SOCS1, induces the stability of SOCS1, and SOCS1 is required for the suppression of p65 by SPTBN1. (A) PLC/PRF/5 and SNU-449 cells were cultured for 48 h and were collected for immunoprecipitation by IgG or antibodies to SOCS1 or SPTBN1. The immunoprecipitates were then analyzed by Western blotting with antibodies to SPTBN1 or SOCS1. (B) SNU-449 cells were transiently transfected with V5-SPTBN1 and HA-SOCS1, V5-SPTBN1 alone or HA-SOCS1 alone for 48 h and the cells were immunoprecipitated by IgG or antibody to V5-epitope. The immune complexes were then subjected to Western blot analysis using antibody to SOCS1. (C) SNU-449 cells were transiently transfected with empty vector or SPTBN1 for 48h. Cells were then treated with cycloheximide (CHX) for the indicated time periods and were analyzed for the stability of SOCS1 and p65 by Western blotting. (D) PLC/PRF/5 cells were transfected as indicated, together with 3 × κB-L reporter plasmid and pRL-TK for 48 h and were lysed to measure luciferase activity. Normalized (to the control siRNA group or vector group) relative luciferase activity is shown (mean ± S.D.). Significance of the difference was evaluated by Student's *t* test (**P < 0.01; ***P < 0.001). (E) SNU-449 cells were transiently transfected with siRNAs without or with HA-SOCS1 plasmid for 48 h. Cells were analyzed by QRT-PCR (left) and harvested cell culture media were analyzed by ELISA (right).

To explore the mechanisms by which SPTBN1 upregulates SOCS1, we used PLC/PRF/5 and SNU-449 cells with SPTBN1 knockdown, as well as liver tissues from WT and *Sptbn1^+/-^* mice, and tested the transcription levels of SOCS1. We observed that the mRNA levels of SOCS1 remained unchanged upon the suppression of SPTBN1 in both PLC/PRF/5 and SNU-449 cells, as well as in mouse liver tissues (data not shown). By analyzing liver hepatocellular carcinoma from TCGA data using GEPIA, we also found there was no correlation between SPTBN1 and SOCS1 at mRNA level (Figure S5A). We then questioned if SPTBN1 functions to stabilize SOCS1 protein, and investigated if SPTBN1 binds to SOCS1. We observed that endogenous SPTBN1 was, in fact, associated with SOCS1 in HCC cells (Figure 5A). The binding of SPTBN1 with SOCS1 was further analyzed by exogenous expression of V5-SPTBN1 and HA-SOCS1 in SNU-449 cells. We observed that SOCS1 was clearly detectable in immunocomplex with an anti-V5 antibody in cells co-transfected with V5-SPTBN1 and HA-SOCS1, but not in cells singly transfected with V5-SPTBN1 or HA-SOCS1 alone (Figure 5B). These data indicate that SPTBN1 is a binding protein to SOCS1.

We further questioned if SPTBN1 is required for stabilization of SOCS1. Overexpression of SPTBN1 was seen to significantly increase the half-life of SOCS1, whereas it shortened the half-life of p65 in cells with overexpressed SPTBN1, compared with cells transfected with empty vector upon the treatment of cycloheximide (CHX) (Figure 5C). It is interesting to note that there was a slight increase in p65 protein levels 5 h after treatment with CHX in SPTBN1 transfected cells, an effect that may be due to a decline in SPTBN1 and SOCS1 proteins after the 5 h treatment period with CHX and the weakened inhibitory effect of SPTBN1, as well as SOCS1, on the p65 protein. The above data indicate that SPTBN1 is a SOCS1 binding protein, required for stabilization of SOCS1, and that SOCS1 is required for the inhibition of p65 by SPTBN1.

We further investigated the impact of SOCS1 on the induction of the NF-κB-dependent luciferase reporter activity of 3×-κB-L, as well as on the production of inflammatory cytokines following inhibition of SPTBN1. Our data indicated that suppression of SPTBN1 led to stimulation of the NF-κB-dependent luciferase reporter activity of 3×-κB-L, and that this induced stimulation was significantly abrogated in cells co-transfected with SPTBN1 siRNA and HA-SOCS1 plasmid (Figure 5D, upper). Further, overexpression of both SPTBN1 and SOCS1 significantly synergized the inhibition of NF-κB-dependent luciferase reporter activity of 3×-κB-L as compared to the effect by overexpression of SPTBN1 or SOCS1 alone (Figure 5D, lower). Additionally, SPTBN1 knockdown-mediated induction of IL-1α, IL-1β, and IL-6 transcription, and secretion of these cytokines into cell culture medium, were abolished by SOCS1 overexpression in HCC cells (Figure 5E). Our data suggest that SOCS1 is a potent mediator of the inflammatory cytokine-suppression function of SPTBN1.

Analysis of the effect of SPTBN1, p65 and SOCS1 on HCC prognosis indicated that HCC patients with low expression of SPTBN1 or SOCS1 is associated with poor survival. However, expression of p65 showed no indication on prognosis for HCC patients (Figure S5B).

### Loss of SPTBN1 promotes the recruitment of immunosuppressive myeloid-derived suppressor cells (MDSCs) and CD4^+^CD25^+^Foxp3^+^ regulatory T cells (Foxp3^+^Tregs) in mouse model

**Figure 6:**
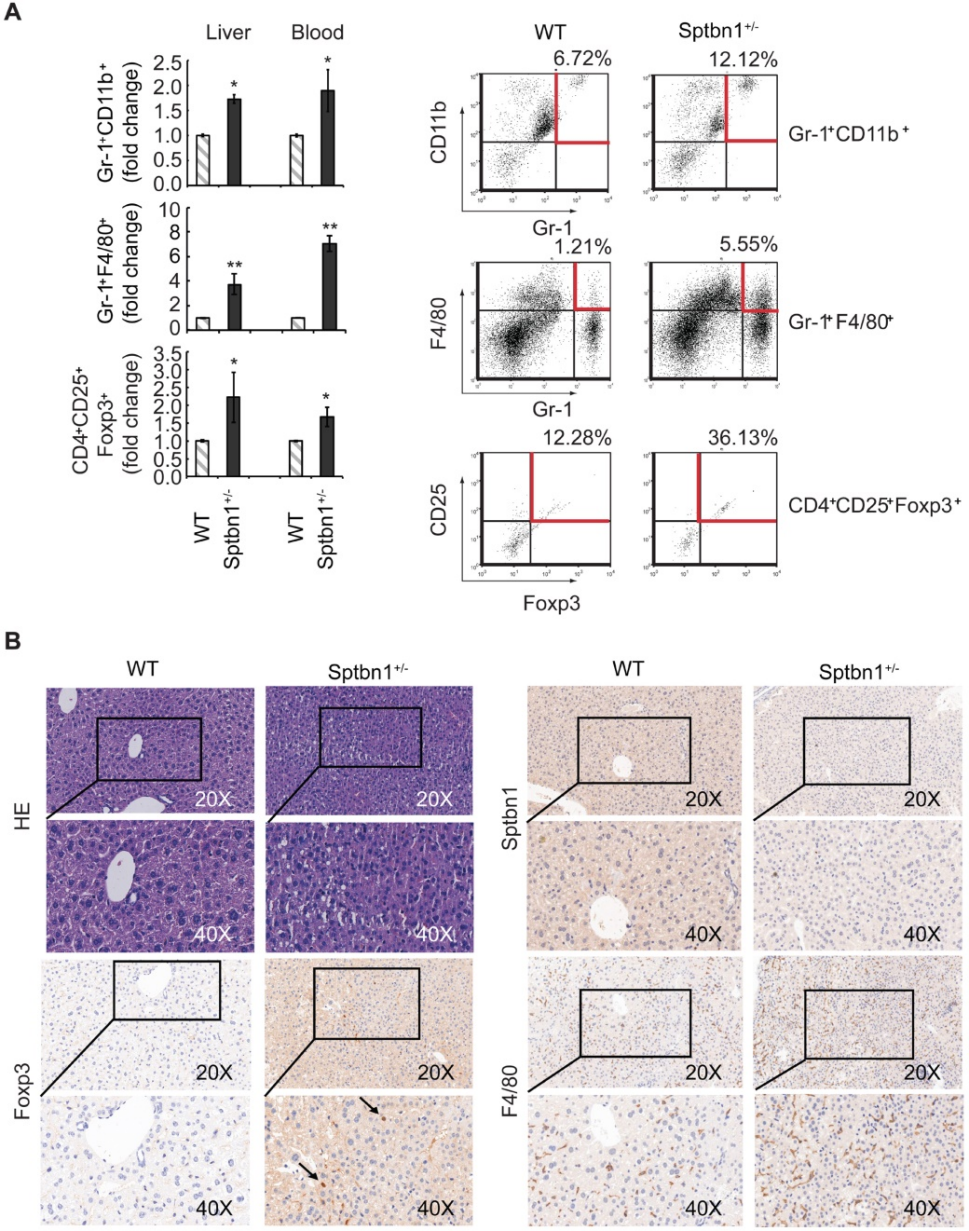
A significant increase in the proportion of MDSCs and Foxp3^+^Treg cells were observed in *Sptbn1^+/-^* mice. (A) FACS analysis of MDSCs and CD4^+^CD25^+^Foxp3^+^Treg cells in CD45-gated single cell suspension of liver and peripheral blood was carried out for WT and *Sptbn1^+/-^* mice (n = 5, aged 6 to 8 months) after staining with antibodies as indicated. The normalized fold-change (mean ± S.D.) (comparing the % of immune cells to WT group) were shown. Significance of differences was evaluated by Student's *t* test (*P < 0.05 and **P < 0.01 versus WT group). Representative FACS blots of MDSCs as defined as Gr-1^+^CD11b^+^, Gr-1^+^F4/80^+^ cells, and Foxp3^+^Treg cells as defined as CD4^+^CD25^+^Foxp3^+^ cells (within areas indicated by horizontal and vertical lines in red color) from WT and *Sptbn1^+/-^* mice liver were shown. (B) Hematoxylin and eosin (HE) and immunohistochemical staining of mouse liver tissues. In liver of *Sptbn1^+/-^* mouse (2 of 4 mice, >15months), well differentiated HCC could be observed. The neoplastic cells grow in a thin trabecular pattern. These cells show mild dysplasia with a slight increase of the nucleus to cytoplasm ratio (HE panels). Numbers of Foxp3-positive Treg cells (arrow pointed) and F4/80-positive macrophages were greater in the cancerous liver of *Sptbn1^+/-^* old mouse than in WT mouse including hepatic sinusoids and perisinusoidal spaces of hepatic lobules.

We then sought to investigate whether the increased expression of inflammatory cytokines seen in *Sptbn1^+/-^* mice was associated with an increase in MDSCs and Foxp3^+^Treg cells, which are reportedly induced by IL-1 and IL-6, and increased in inflammatory pathological conditions 38-43. To answer this question, we used multiparameter flow cytometric analysis to quantify the accumulation of MDSCs and Foxp3^+^Treg cells in liver, spleen, bone marrow, and peripheral blood from WT and *Sptbn1^+/-^* mice. We observed a significantly elevated accumulation of immune suppressive MDSCs (Gr-1^+^CD11b^+^, *P* < 0.05, and Gr-1^+^F4/80^+^, *P* < 0.01) and Foxp3^+^Treg cells (CD4^+^CD25^+^Foxp3^+^, *P* < 0.05) in the liver and peripheral blood (but not the spleen or bone marrow) of *Sptbn1^+/-^* mice, compared with age- and gender- matched WT mice (Figure 6A).

In addition, in liver of *Sptbn1^+/-^* mouse (2 of 4 mice, > 15months), well differentiated HCC could be observed. The neoplastic cells grow in a thin trabecular pattern and show mild dysplasia with a slight increase of the nucleus to cytoplasm ratio. Sinusoid-like blood spaces show varying degrees of dilation in some area, while in some other area, are difficult to recognize due to compression of tumor cells (Figure 6B, HE panels). Numbers of Foxp3-positive Treg cells (arrow pointed) and F4/80-positive macrophages were greater in the cancerous liver of *Sptbn1^+/-^* old mouse than in WT mouse including hepatic sinusoids and perisinusoidal spaces of hepatic lobules (Figure 6B).

### Loss of SPTBN1 promotes hepatocarcinogenesis induced by DDC through NF-κB (p65) signaling pathway

**Figure 7:**
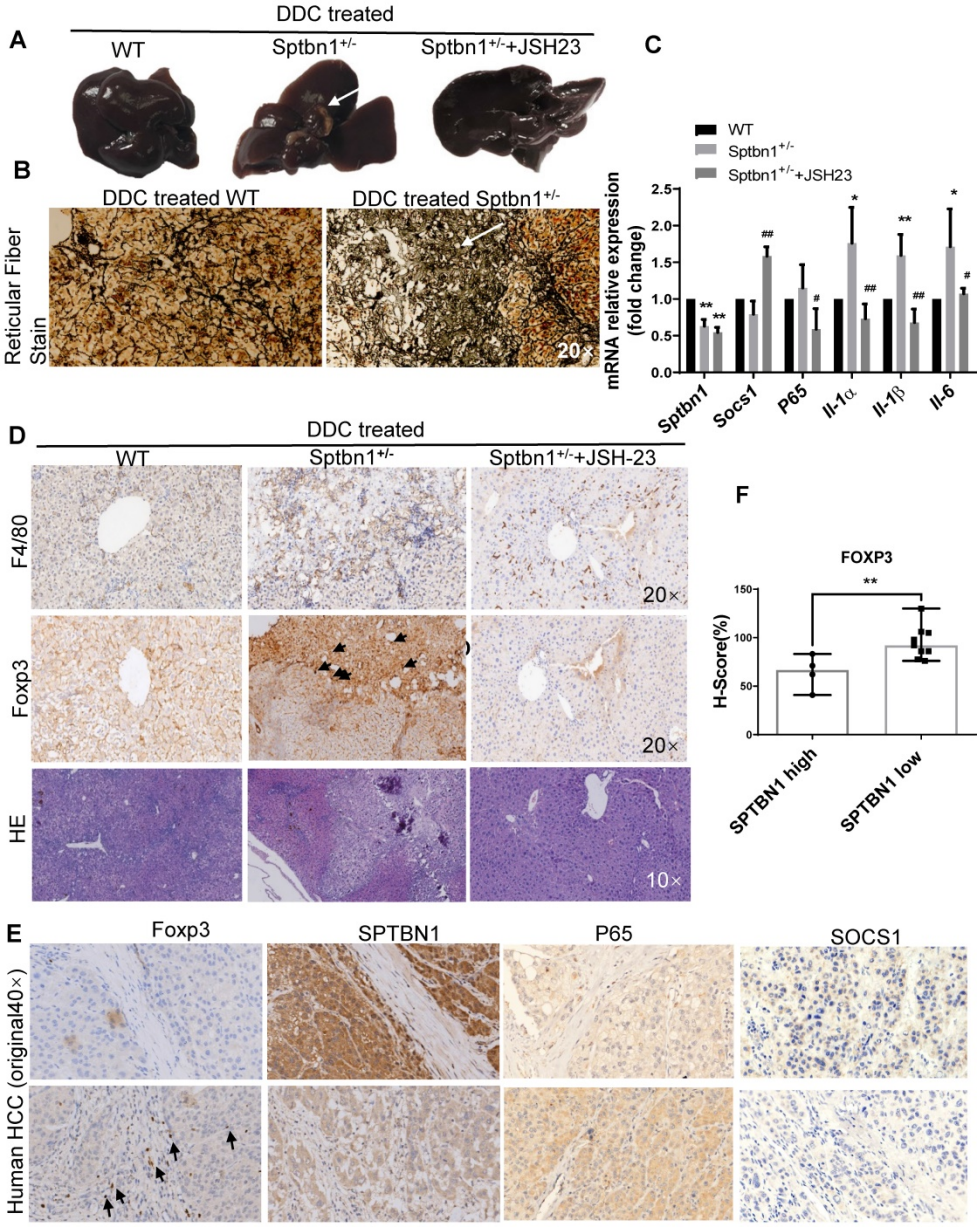
Loss of SPTBN1 promotes hepatocarcinogenesis and inflammatory through p65 signaling pathway. A. Upon treatment with 0.1% DDC-containing diet for 3 months, Sptbn1^+/-^ mice developed HCC (arrows), compared to WT and JSH-23 (p65 inhibitor) treated *Sptbn1^+/-^* mice. B. Liver tissues from WT and *Sptbn1^+/-^* mice treated with 0.1% DDC for 3 months were analyzed by Gomori's reticular fiber staining. C. Liver tissues from WT and *Sptbn1^+/-^* mice treated with 0.1%DDC in the absence or presence of JSH-23 for 3 months were analyzed by QRT-PCR. The mRNA levels of IL-1α, IL-1β, IL-6, SOCS1 and p65 were detected. *P < 0.05, **P < 0.01 (WT vs *Sptbn1^+/-^*, n = 4). D. IHC and H&E staining. Liver tissues from mice treated same as in Figure 7A were analyzed by immunohistochemistry staining by antibodies targeting macrophages and Treg cells. E. Human HCC tissues were analyzed by immunohistochemistry staining by antibodies against FOXP3, p65, SOCS1 and SPTBN1. F. FOXP3 staining was evaluated by immunohistochemistry score in human HCC. H-score was determined based on the intensity of nuclear staining and the proportion of labeled tumor cells. **P < 0.01 vs SPTBN1 high. n = 13.

In addition to the spontaneous liver cancer formation in mice with impaired SPTBN1, we also studied whether *Sptbn1^+/-^* mice are susceptible to the DDC induced malignancy in liver. The results showed that in the livers of mice fed with 0.1% DDC -containing diet for 3 months, 3 of 7 *Sptbn1^+/-^* mice developed liver cancer while there is no tumor visible in WT mice and *Sptbn1^+/-^* mice treated with NF-κB inhibitor JSH-23 (Figure 7A and Figure S5C). Pathological indication of hepatocellular carcinoma formation and degradation or collapse of reticular fibers could be observed in the livers from *Sptbn1^+/-^* mice treated with DDC-containing diet (Figure 7B). Further analysis indicated that, compared to livers from DDC treated WT and combination of DDC with NF-κB inhibitor treated *Sptbn1^+/-^* mice, IL-1α, IL-1β and IL-6 mRNA levels were increased in tumor burden livers from DDC treated *Sptbn1^+/-^* mice (n = 3) (Figure 7C), which clearly demonstrate that the increased pro-inflammatory cytokines upon suppression of SPTBN1 were p65-dependent. Co-immunofluorescence staining showed that SOCS1 expression was decreased while nuclear p65 was increased in the cancerous liver from *Sptbn1^+/-^* mice after treatment with DDC, and p65 inhibition by JSH-23 reversed these effects resulted from SPTBN1 suppression (Figure S5D), which was also demonstrated in PLC/PRF/5 cells (Figure S2).

We further analyzed infiltration of macrophages and Treg cells in liver cancers of mice as well as in human HCC tissues upon impairment of SPTBN1 by IHC. As shown in Figure 7D, increased F4/80-positive macrophages and Foxp3-positive Tregs could be observed in the cancerous livers from *Sptbn1^+/-^* mice induced after feeding with DDC-containing diet but not in *Sptbn1^+/-^* mice treated with combination of DDC with p65 inhibitor or in WT mice with DDC-containing diet. In human HCC (n = 13), staining of Foxp3-positive Tregs was greater in HCC with lower SPTBN1 expression (Figure 7E-F). We also found that the expression of SOCS1 was hardly detectable while p65 was higher in human HCC with lower SPTBN1 expression (Figure 7E).

Furthermore, we questioned whether inhibition of p65 by either p65 inhibitor JSH-23 or p65 siRNA has effect on the expression of SPTBN1 or SOCS1 in the circuit of abrogation of induced expression of p65 upon suppression of SPTBN1. We found that treatment with p65 inhibitor JSH-23 in the system leading to significant increase of mRNA levels of SOCS1 but not SPTBN1 in mice livers and SNU-449 cells (Figure 7C, Figure S6A), however, treatment with p65 siRNA did not reach statistical significance in the feedback induction of SOCS1 gene in SNU-449 cells (Figure S6B).

## Discussion

In our previous study, we have shown that loss of SPTBN1 in PLC/PRF5 and SNU449 HCC cells increased the abilities of sphere formation, xenograft tumor development and invasion, indicating that SPTBN1 is an essential suppressor of tumorigenesis 10. In this report, we demonstrate that SPTBN1 is a binding protein of SOCS1, and functions to suppress inflammatory cytokine expression via the inhibition of p65, one of the strongest activators of the NF-κB family. Additionally, we show that heterozygous loss of SPTBN1 (*Sptbn1^+/-^*) in mice leads to an increase in the proportion of immunosuppressive MDSCs (Gr-1^+^CD11b^+^ and Gr-1^+^F4/80^+^) and Foxp3^+^Treg (CD4^+^CD25^+^Foxp3^+^) cells in the liver and peripheral blood. And that loss of SPTBN1 promotes DDC-induced hepatocarcinogenesis with increased inflammatory microenvironment through p65 signaling pathway.

It is known that NF-κB transcription factors control the transcription of genes that regulate innate and adaptive immune responses. Uncontrolled regulation of NF-κB is linked to disease states such as chronic inflammation and cancer 44. The regulation of NF-κB by IκB is well recognized as one of the major regulatory mechanisms that modulate NF-κB activity 16, 21, 22, 45. Studies also indicate that an IκBα-independent termination of NF-κB signal transduction by ubiquitin- and proteasome-dependent degradation of the p65 subunit in the nucleus is critical for the efficient termination of NF-κB activation 22, 45. We found that suppression of SPTBN1, as well as heterozygous loss of SPTBN1 (*Sptbn1^+/-^*), in HCC cells and mouse liver cells leads to the accumulation of p65 within the nucleus of these cells. It does not appear that the nuclear retention of p65 is caused by phosphorylation and degradation of its upstream inhibitor IκBα because we observed no difference in the level of IκBα phosphorylation in HCC cells upon inhibition of SPTBN1 expression. Instead, we detected enhanced expression of IκBα in HCC cells in tandem with SPTBN1 suppression, which is consistent with the known negative feedback loop involving an increase in endogenous IκBα protein with overexpression of p65 by mechanisms of protein stabilization and increased IκBα mRNA 46. Likewise, our analysis in human HCC cells indicates that suppression of SPTBN1 causes nuclear accumulation of p65 due to increased p65 protein stability.

Our results suggest that SPTBN1 controls NF-κB activation by reducing the protein level of p65 through ubiquitination and subsequent proteasome-dependent degradation of p65. Protein ubiquitination is a fundamental regulatory posttranslational modification reaction that controls intracellular signaling events 47. This process involves three sequential steps: activation, conjugation, and ligation, which are carried out by ubiquitin-activating enzymes (E1s), ubiquitin-conjugating enzymes (E2s), and ubiquitin ligases (E3s). The reaction catalyzed by each enzyme results in the binding of ubiquitin to lysine residues on the target protein substrate. The sensitivity and specificity of this protein substrate labeling with ubiquitin during these three catalytic steps is determined mainly by E3 ligases 47. Among the identified putative E3 ligases of p65, including SOCS1, PIN1, COMMD1, and PPARγ, it was SOCS1 that was regulated by SPTBN1 in PLC/PRF/5 and SNU-449 cells, and *Sptbn1^+/-^* mouse liver tissues 34-37. SOCS1 belongs to the SOCS protein family, a group of inhibitory regulators of numerous cytokine-signaling pathways that play a pivotal role in maintaining organ homeostasis 48. SOCS1 is a p65-binding protein that functions as a ubiquitin ligase of p65, causing polyubiquitination and proteasome-dependent degradation of nuclear p65 34, 35. In liver tissues from age- and gender-matched *Sptbn1^+/-^* and WT mice, we observed that the nuclear expression levels of p65 and SOCS1 were inversely correlated in *Sptbn1^+/-^* mice; that is, SOCS1 was significantly downregulated while p65 was significantly upregulated in *Sptbn1^+/-^* mice compare to WT mice. Our study in HCC cells revealed that overexpression of SOCS1 abrogated the increased expression of p65 caused by inhibition of SPTBN1, demonstrating that SOCS1 is required for the regulation of p65 by SPTBN1.

We revealed that endogenously and exogenously expressed SPTBN1 and SOCS1 were associated with each other in HCC cells, and that the binding of SPTBN1 with SOCS1 appeared to intensify the stability of the SOCS1 protein by SPTBN1. SOCS1 is purportedly important in various liver diseases 48. SOCS1 deficient mice die at or before three weeks of age due to hepatic inflammation caused by aberrant activation of T cells, fatty degeneration, and hepatocyte necrosis 49, 50. Clinical studies reveal that frequent CpG island methylation of the SOCS1 gene is observed in human primary hepatocellular carcinomas (HCCs) 51. In human HCC tissues, we also found that SOCS1 could be weakly stained in HCC with higher SPTBN1 expression and hardly detectable in HCC with lower SPTBN1 expression, which is consistent with the report that the incidence of SOCS1 aberrant methylation was 65% in human primary HCC tumor samples 51. These findings indicate that SOCS1 acts as a suppressor of hepatic inflammation and development of hepatocellular carcinomas. Based on findings presented thus far from others, the downregulation of SOCS1, and upregulation of nuclear expression of p65 seen in HCC cells upon the suppression of SPTBN1 might be a critical molecular event that promotes inflammatory cytokine expression, as addressed by our present results.

We further observed that suppression of SPTBN1 (*Sptbn1^+/-^* mice) is associated with increased recruitment of MDSCs and Foxp3^+^Treg cells in liver and peripheral blood compared with WT mice. MDSCs are a heterogeneous population of early myeloid progenitors, which include immature granulocytes, macrophages, and dendritic cells at different stages of differentiation. These myeloid progenitors can be identified in mice by the expression of Gr-1 and CD11b on recognized granulocytic and monocytic subsets 39, 40. MDSCs exert tumor-promoting capacity via the potent inhibition of T cell and NK cell proliferation and activation to eliminate anti-tumor immunity and facilitate tumor angiogenesis and metastasis 39, 40. IL-1 and IL-6 are key factors that promote the recruitment and expansion of MDSCs 41-43. Stomach-specific expression of human IL-1β in mice induces gastric inflammation and cancer, with recruitment of MDSC in the stomach, and that NF-κB signaling is involved in this process 42. The induction of CD4+CD25+Foxp3+Treg cells appears to contribute to the formation of immune-suppressive conditions that facilitate tumor immune escape mechanisms 52. Results from another clinical study indicate that MDSC induces CD4^+^CD25^+^Foxp3^+^Treg cells in HCC patients 53. In PTEN null mice, a massive infiltration of CD11b^+^Gr-1^+^ myeloid cells in a prostate tumor site can protect a fraction of tumor cells from senescence 54. The induction of MDSCs and Foxp3^+^Treg cells, as well as the induction of inflammatory cytokines following the loss of SPTBN1 in mice and HCC cells, suggest that inflammatory-associated immune-suppressive conditions may be a critical mechanism for the formation and progression of liver cancer caused by suppression or deletion of SPTBN1; this theory merits further comprehensive and systematic exploration. Our present observations may certainly contribute to the future understanding of the tumor suppressor function of SPTBN1 in cancer immune evasion, and may provide a model for testing therapeutic agents against the NF-κB-mediated inflammatory responses and eventually overcome cancer immune escape.

Our work has also indicated that in the cohort of HCC patients with *SPTBN1* low/*IL-1β* high, or *SPTBN1* low/*IL-6* high, the loss of *SPTBN1* and increased level of *IL-1β* or *IL-6* correlates with shorter relapse-free survival, which highlights *SPTBN1*, *IL-1β,* and *IL-6* as prognostic biomarkers for clinical outcome in HCC patients. This warrants further investigation of these molecular entities as predictive biomarkers and for developing therapeutic agents against the NF-κB-mediated inflammatory response in HCC patients.

**Figure 8:**
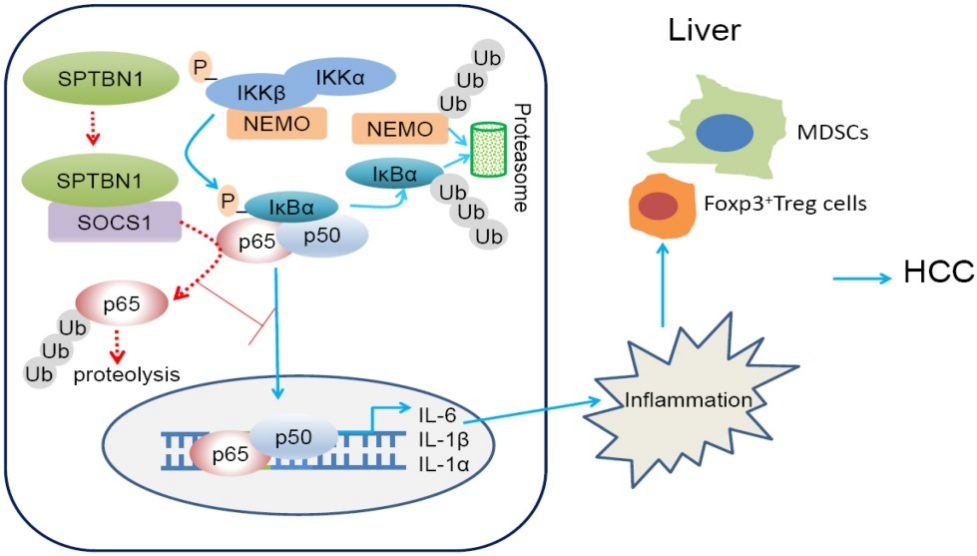
Graphical abstract. Loss of SPTBN1 increases p65 protein stability via the inhibition of SOCS1 and enhances NF-κB activation, stimulating inflammatory responses and immune-suppressive conditions for the formation and progression of liver cancer.

In summary (Figure 8, graphical abstract), we reveal that SPTBN1 functions as a tumor suppressor to stabilize SOCS1 protein, controlling the cellular level of p65 to suppress the expression of inflammatory cytokines, IL-1α, IL-1β and IL-6, and the expansion of MDSCs and Foxp3^+^Treg cells. Through this process, SPTBN1 thereby inhibits inflammatory responses and immune-suppressive conditions, which have been demonstrated extensively to be highly associated with cancer formation and progression.

## Significance

Loss of SPTBN1 increases p65 protein stability via the inhibition of SOCS1 and enhances NF-κB activation, stimulating inflammatory responses and immune-suppressive conditions for the formation and progression of liver cancer.

## Abbreviations

SPTBN1: Spectrin, beta, non-erythrocytic 1
TGF-β: transforming growth factor beta
HCC: hepatocellular carcinoma
IL-1α: interleukin-1α
IL-1β: interleukin-1β
IL-6: interleukin-6
SOCS1: suppressor of cytokine signaling 1
MDSC: myeloid-derived suppressor cell
Treg cell: regulatory T cell
