# Supplementary material for: SPTBN1 inhibits inflammatory responses and hepatocarcinogenesis via the stabilization of SOCS1 and downregulation of p65 in hepatocellular carcinoma
Source: Theranostics. 2021 Feb 20;11(9):4232–50. doi: 10.7150/thno.49819 (PMC7977457; doi:10.7150/thno.49819)
Supplement: Supplementary file 1 — Supplementary figures and tables. [file thnov11p4232s1.pdf]

Supplementary Materials

Methods

Investigating the clinical relevance of genes with survival

We studied the clinical relevance of SPTBN1, IL-1β, IL-6 and IL-1α gene expression with relapse-free survival using the human HBV-induced HCC gene array database GSE14520 (the HCV-induced HCC dataset did not have survival data and hence could not be used for this analysis). We wanted to investigate how the combination of *SPTBN1* and *IL-1β/IL-6/IL-1α* affected relapse-free survival. We applied Cox proportional hazards model using statistical analysis tool R and investigated the gene expression ratio of (a) SPTBN1/IL-1β (b) IL-1β/SPTBN1, and (c) SPTBN1/IL-6 (1, 2). We also investigated other gene expression ratios IL-6/SPTBN1, SPTBN1/IL-1α, IL-1α/SPTBN1, these results were not significant, and hence are not detailed in this document.

The original microarray gene expression data was in log base 2 numerical format. The log inverse of these values were taken for this analysis. For example, if "A" be the log base 2 gene expression value of GeneA. "B" is the log base 2 gene expression value of GeneB
Formula used in the model: **C + D + C * D + E**

where:

C = log inverse of GeneA

D = log inverse of GeneB

E = actual gene expression ratio of GeneA and GeneB = C/D

C * D (also denoted as C:D) is the interaction term

(a) **Investigating the clinical relevance of gene expression ratio of SPTBN1/ IL-1β**
Among the 212 tumor samples analyzed in the GSE14520 (human HBV-induced HCC) study, 72 HCC samples had the expression of *SPTBN1* below the median level among the whole group of samples and expression of *IL-1β* above the median level among the whole group (*SPTBN1* low/*IL-1β* high). This was the sub-group of samples chosen for this analysis.

The formula used in the model in R is shown below. The output of the model is in terms of the coefficients of the model and the corresponding confidence intervals (Supplementary Table A). Other outputs measures of the model that indicate the fit include Concordance, and Rsquare value. The model also outputs its overall p-value from Likelihood ratio test, Wald test and Score (logrank) tests shown below in red font as significant (p value less than 0.05).

*Coxph(formula = obj.surv ~ SPTBN1 + IL-1β + SPTBN1 * IL-1β + SPTBN1/IL-1β , method = "breslow")*

N = 72, number of events = 41

Concordance = 0.642 (se = 0.048)
Rsquare = 0.126 (max possible = 0.988)
Likelihood ratio test = 9.72 on 4 df, p = 0.04537
Wald test = 10.08 on 4 df, p = 0.03916
Score (logrank) test = 10.89 on 4 df, p = 0.02779

From this model, we can interpret the following:
Holding all other covariates constant, we see that unit increase in the actual gene expression ratio of SPTBN1/IL-1β, decreases the hazard of event (progression in disease) by a factor of 0.634 (which is about 36.6%) in the *SPTBN1*-Low and *IL-1β*-high subgroup of patients.

We already know from Figure 1B that there is an inverse correlation between *1L-1β* and *SPTBN1*. So if the expression of *SPTBN1* increases and/or the expression of *IL-1β* decreases, the ratio *SPTBN1*/*IL-1β* increases, which in-turn decreases the hazard of an event. Alternatively, a decrease in expression of *SPTBN1* and/or increase in expression of *IL-1β* causes the ratio of *SPTBN1*/*IL-1β* to decrease, which in-turn increases the hazard of an event.

Table S1: Coefficients and confidence intervals of the model

Explanation	coef	exp(coef)	se(coef)	z	exp(-coef)	lower .95	upper .95	Pr(> |z|)
SPTBN1	0.057	1.058	0.042	1.364	0.945	0.976	1.148	0.172
IL-1β	0.066	1.068	0.098	0.666	0.937	0.881	1.295	0.505
SPTBN1/IL-1β	-0.455	0.634	0.296	-1.538	1.577	0.355	1.133	0.124
SPTBN1 : IL-1β interaction term	-0.001	0.999	0.001	-0.817	1.001	0.996	1.002	0.414

coef: coefficient (beta) of the model ; exp(coef): Hazard Ratio; se(coef): Standard Error; lower and upper 0.95: 95% confidence intervals; Pr(> |z|): p-value;

(b) Investigating the clinical relevance of gene expression ratio of IL-1β/SPTBN1

Among the 212 tumor samples analyzed in the GSE14520 (human HBV-induced HCC) study, 72 HCC samples had the expression of *SPTBN1* below the median level among the whole group of samples and expression of *IL-1β* above the median level among the whole group (*SPTBN1* low/*IL-1β* high). This was the sub-group of samples chosen for this analysis.

The formula used in the model in R is shown below. The output of the model is in terms of the coefficients of the model and the corresponding confidence intervals (Supplementary Table B). Other outputs measures of the model that indicate the fit include Concordance, and Rsquare value. The model also outputs its overall p-value from Likelihood ratio test, Wald test and Score (logrank) tests. Those that are significant (p value less than 0.05) are shown in red font.

*Coxph(formula = obj.surv ~ IL-1β + SPTBN1 + IL-1β * SPTBN1 + IL-1β/ SPTBN1 , method = "breslow")*

n = 72, number of events = 41

Concordance = 0.615 (se = 0.048)

Rsquare = 0.103 (max possible = 0.988)

Likelihood ratio test = 7.82 on 4 df, p = 0.09821

Wald test = 9.41 on 4 df, p = 0.05171

Score (logrank) test = 10.34 on 4 df, p = 0.03507

Table S2: Coefficients and confidence intervals of the model

Explanation	coef	exp(coef)	se(coef)	z	exp(-coef)	lower .95	upper .95	Pr(> |z|)
IL-1β	-0.087	0.917	0.088	-0.990	1.091	0.772	1.089	0.322
SPTBN1	-0.005	0.995	0.009	-0.563	1.005	0.977	1.013	0.574
IL-1β/ SPTBN1	2.504	12.231	3.255	0.769	0.082	0.021	7219.357	0.442
IL-1β : SPTBN1 interaction tern	0.001	1.001	0.001	1.625	0.999	1.000	1.002	0.104

coef: coefficient (beta) of the model ; exp(coef): Hazard Ratio; se(coef): Standard Error; lower and upper 0.95: 95% confidence intervals; Pr(> |z|): p-value;

From this model, we can interpret the following: Holding all other covariates constant, we see that unit increase in the actual gene expression ratio of *IL-1β/SPTBN1* increases hazard of event (progression of disease) by a factor of 12 (which is about 1123%) in the *SPTBN1*-Low and *IL-1β*-high subgroup of patients.

We already know that there is an inverse correlation between *IL-1β* and *SPTBN1*. So if the expression of *IL-1β* increases, and/or the expression of *SPTBN1* decreases, the ratio *IL-1β/SPTBN1* also increases, which in-turn increases the hazard of an event.

(c) Investigating the clinical relevance of gene expression ratio of SPTBN1/IL-6

Out of 212 tumor samples in this study, 41 human HBV-induced HCC samples had the expression of *SPTBN1* below the median level among the whole group of samples and expression of *IL-6* above the median level among the whole group (*SPTBN1* low/*IL-6* high). This was the sub-group of samples chosen for this analysis.

The formula used in the model in R is shown below. The output of the model is in terms of the coefficients of the model (Supplementary Table C1) and the corresponding confidence intervals (Supplementary Table C2). Other outputs measures of the model that indicate the fit include Concordance, and Rsquare value. The model also outputs its overall p-value from Likelihood ratio test, Wald test and Score (logrank) tests. Those that are significant (p value less than 0.05) are shown in red font.

Coxph(formula = obj.surv ~ SPTBN1 + IL-6 + SPTBN1 IL-6 + SPTBN1/ IL-6 , method = "breslow")*

n = 41, number of events = 21

Concordance = 0.674 (se = 0.068)

Rsquare = 0.194 (max possible = 0.967)

Likelihood ratio test = 8.84 on 4 df, p = 0.06534

Wald test = 9.54 on 4 df, p = 0.04901

Score (logrank) test = 11.33 on 4 df, p = 0.0231

Table S3: Coefficients and confidence intervals of the model

Term	coef	exp(coef)	se(coef)	z	exp(-coef)	lower .95	upper .95	Pr(>|z|)
SPTBN1	1.413	4.109	4.157	0.340	0.243	0.001	14202.377	0.734
IL-6	-0.171	0.843	2.886	-0.059	1.186	0.003	241.379	0.953
SPTBN1/ IL-6	-9.042	0.000	4.994	-1.811	8449.970	0.000	2.109	0.070
SPTBN1: IL-6 interaction tern	0.003	1.003	0.325	0.008	0.997	0.530	1.897	0.993

coef: coefficient (beta) of the model ; exp(coef): Hazard Ratio; se(coef): Standard Error; lower and upper 0.95: 95% confidence intervals; Pr(> |z|): p-value;

From this model, we can interpret the following:

Holding all other covariates constant, we see that unit increase in the actual gene expression ratio of *SPTBN1*/*IL-6* decreases hazard of event (progression of disease) by a factor of 0 (which is 100%) in the *SPTBN1*-Low and *IL-6*-high subgroup of patients.

So if either the expression of *SPTBN1* increases or the expression of *IL-6* decreases, the ratio *SPTBN1*/ *IL-6* increases, which in-turn decreases the hazard of an event. Alternatively, a decrease in expression of *SPTBN1* or increase in expression of *IL-6* causes the ratio of *SPTBN1*/*IL-6* to decrease, which in-turn increases the hazard of an event.

(d) Investigating the the clinical relevance of gene expression ratio of SPTBN1/two or three above cytokins

Table S4：Clinical relevance of SPTBN1/two or three above cytokins

GSE14520 HBV HCC cohort（Total = 212 patients）				
**Genes**	**Number of patients**	**%**	**Overall survival status (0 means alive/censoring, 1 means dead)**	**Recurr survival status (0 means recur/censoring, 1 means recurrence)**
* SPTBN1 low/IL-1α high	63	30%	41 patients status 0; 22 patients status 1	31 patiens with Status 0; 32 patients with Status 1
* two or all three cytokines high/ SPTBN1 low				
(a) IL-1α high, IL-1β high, IL-6 high and SPTBN1 low	36	17%	24 patients status 0; 12 patients status 1	19 patiens with Status 0; 17 patients with Status 1
(b) IL-1α high, IL-1β high and SPTBN1 low	45	21%	27 patients status 0; 18 patients status 1	20 patiens with Status 0; 25 patients with Status 1
(c) IL-1α high, IL-6 high and SPTBN1 low	47	22%	35 patients status 0; 12 patients status 1	28 patiens with Status 0; 19 patients with Status 1
(d) IL-1β high, IL-6 high and SPTBN1 low	54	25%	34 patients status 0; 20 patients status 1	26 patiens with Status 0; 28 patients with Status 1

Supplementary Figure

Figure S1

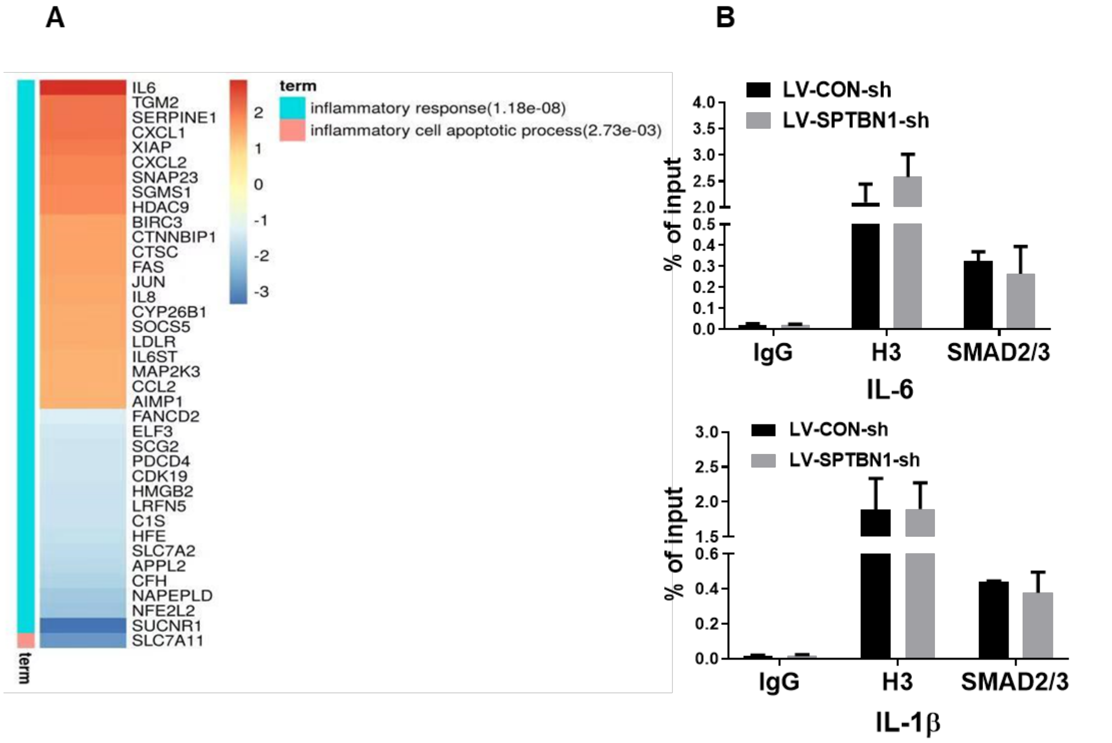

Figure S1.**A**. The heat map showed differential expression of inflammatory genes in SNU449 cells with or without SPTBN1 knockdown. **B**. ChIP-qPCR was performed in LV-SPTBN1sh-infected PLC/PRF/5 cells with anti-Smads2/3 antibodies to determine the enrichment of promoter region sequences of IL-6(upper) and IL-1β (lower) in the obtained ChIP DNA.

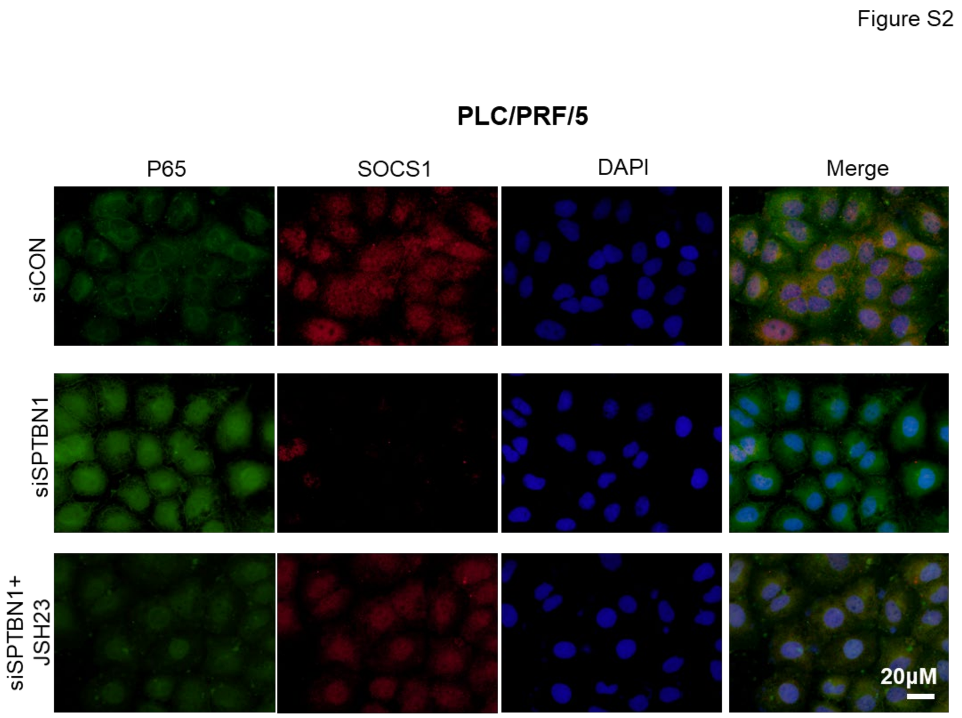

Figure S2.PLC/PRF/5 cells treated with control siRNA, SPTBN1 siRNA or SPTBN1 siRNA plus p65 inhibitor JSH-23 were analyzed by co-immunofluorescence staining for SOCS1 and p65.

Figure S3

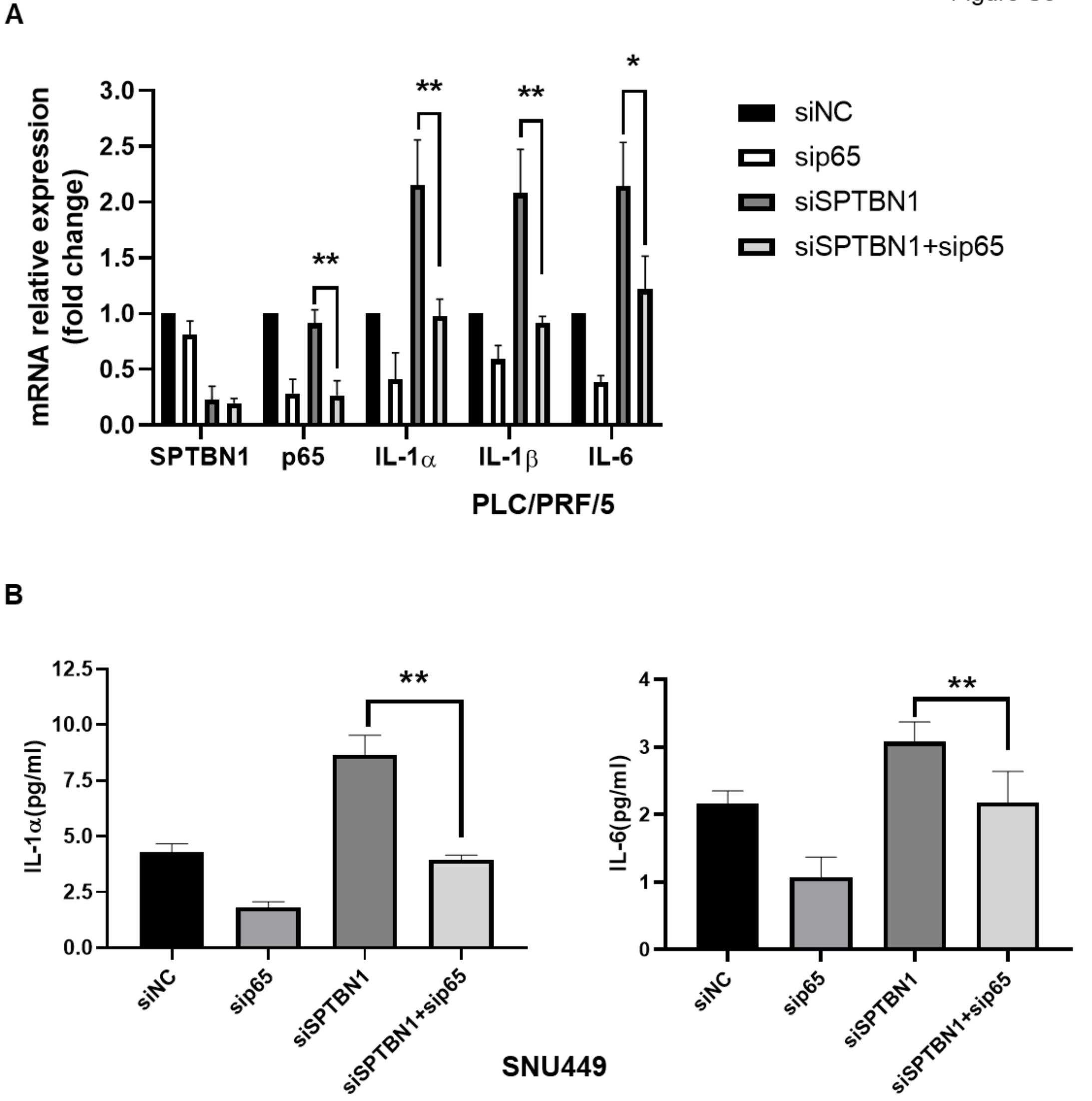

Figure S3.**A**. PLC/PRF/5 cells were transiently transfected with siRNAs as indicated for 48 h. Cells were then analyzed by QRT-PCR for mRNA levels of SPTBN1, p65, IL-1α, IL-1β and IL-6. **B.** SNU-449 cells were transiently transfected with siRNAs as indicated for 48 h. Harvested cell culture media were analyzed by ELISA.

Figure S4

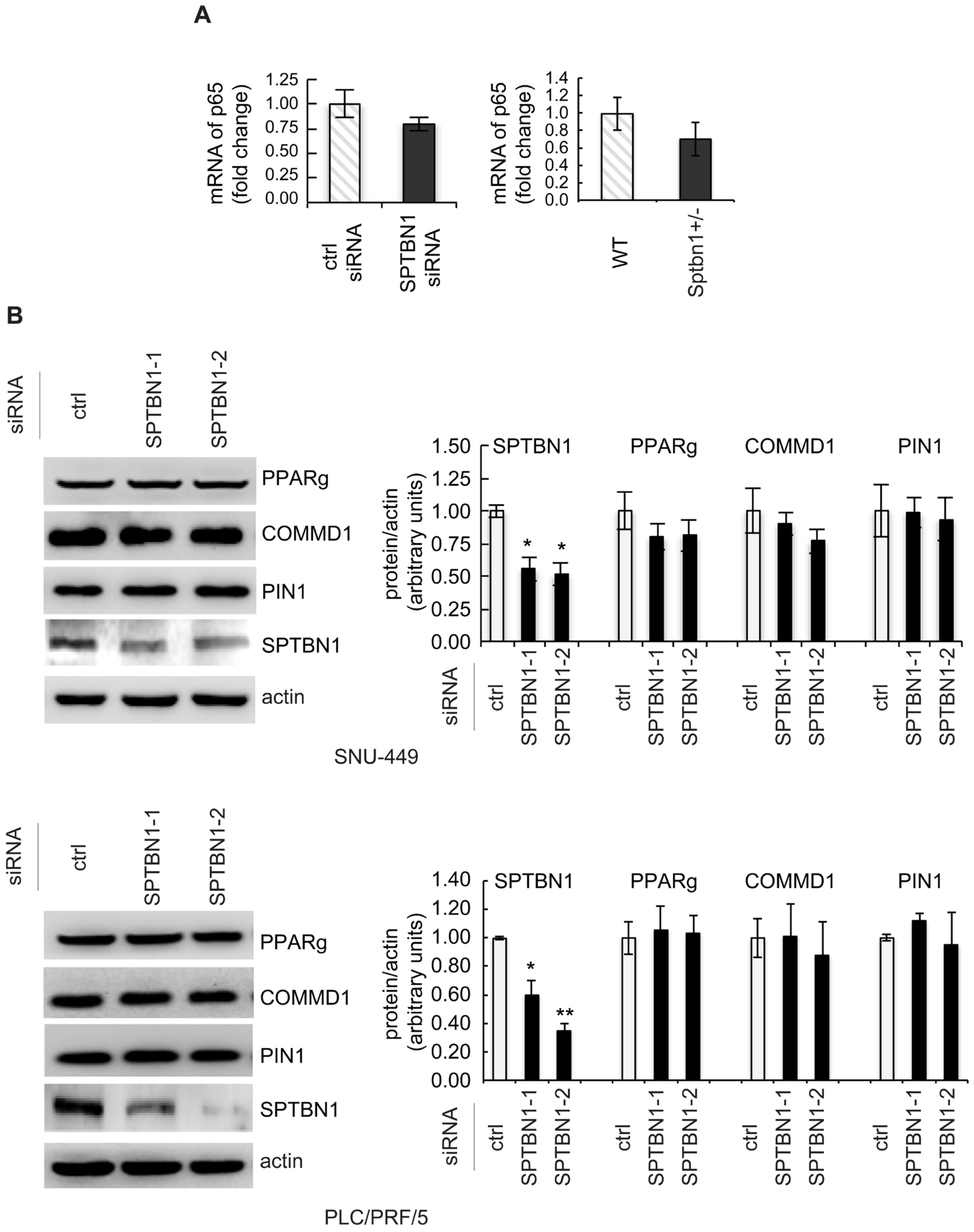

Figure S4.**A**. QRT-PCR analysis of p65 mRNA in PLC/PRF/5 cells after transient transfection with control siRNA or siRNA to SPTBN1 (left) and in liver tissues from WT and *Sptbn1*$^{+/-}$ mice (right). **B.** SNU-449 (upper) and PLC/PRF/5 (lower) cells were

transfected with control siRNA or siRNA to SPTBN1 for 48 hours and the cells were analyzed by Western blot for the proteins as indicated. The relative intensities of COMMD1, PIN1, PPARγ to actin in two to three independent Western blotting were analyzed as in Figure 2A and 2B. $^{*}P < 0.05$, $^{**}P < 0.01$ vs ctrl, n = 3

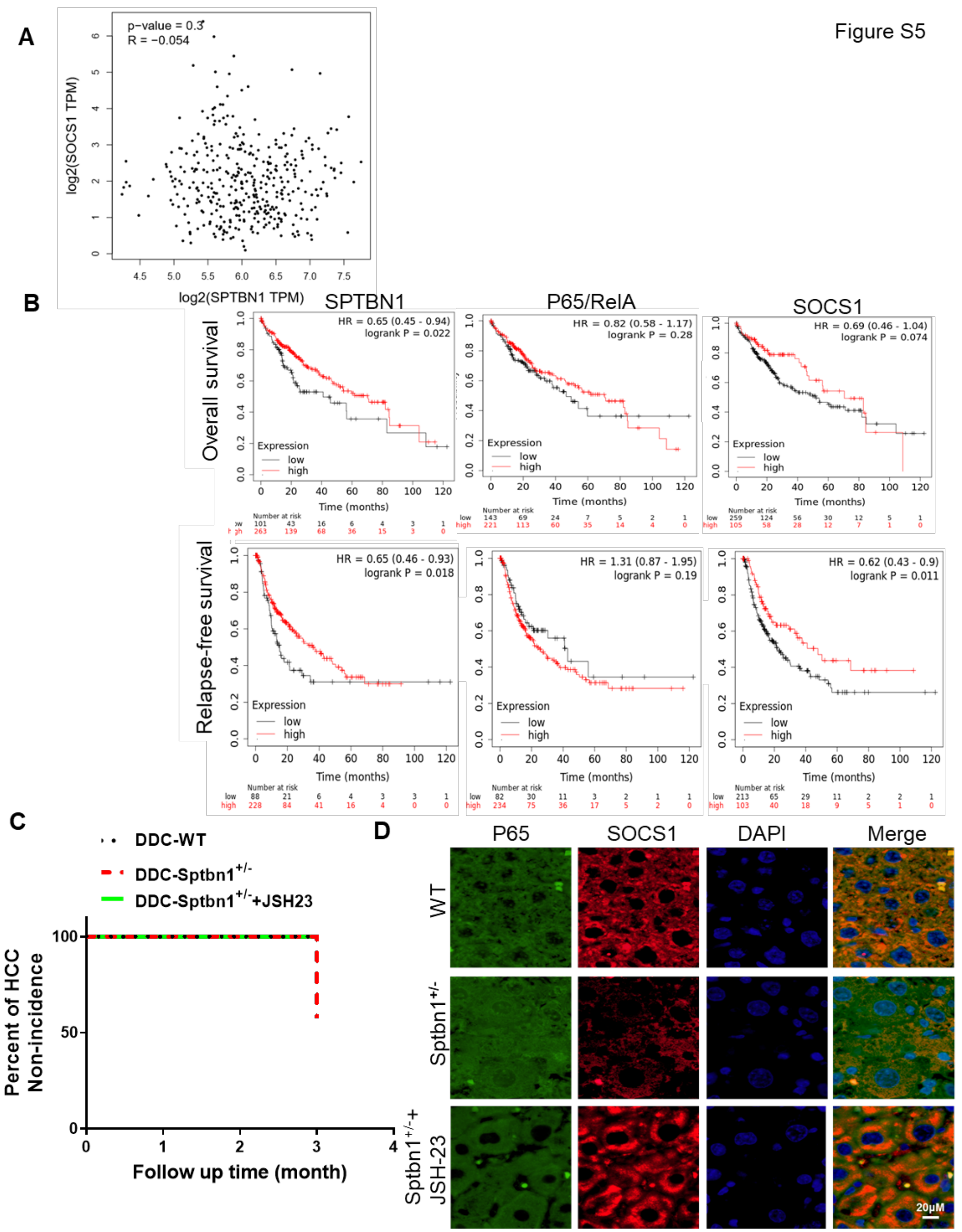

Figure S5.**A**. The expression correlation between SPTBN1 and SOCS1 was analyzed using TCGA database by GEPIA (http://gepia.cancer-pku.cn/detail.php?clicktag=correlation). **B**. The effect of SPTBN1, p65 and SOCS1 in the

overall survival (OS) and relapse free survival (RFS) of patients with HCC was analyzed by Kaplan-Meier-plotter. **C**. Rate of HCC non-incidence. After treatment with 0.1% DDC-containing diet for 3 months, *Sptbn1*$^{+/-}$ mice exhibited an increased prevalence of HCC, compared to WT mice or *Sptbn1*$^{+/-}$ mice treated p65 inhibitor JSH-23 simultaneously. **D**. Liver tissues from mice groups as described in S5-C were analyzed by co-immunofluorescence staining for SOCS1 and p65. JSH-23 treatment partially reversed the phenotype of *Sptbn1* knockdown.

Figure S6

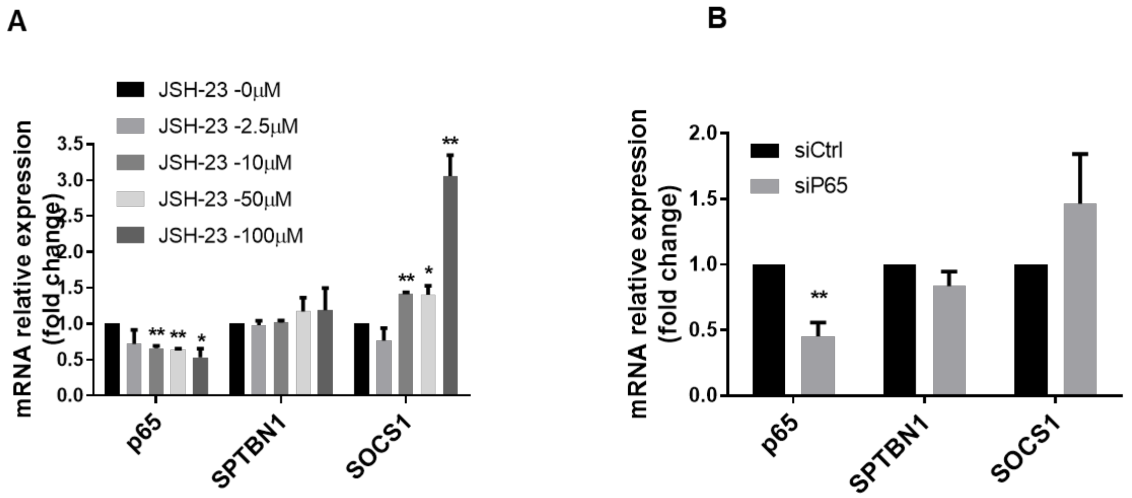

Figure S6.**A**. The mRNA levels of SPTBN1, SOCS1 and p65 were analyzed by QRT-PCR in SNU-449 cells treated with different concentration of JSH-23 for 24 h, $^{*}P < 0.05$, $^{**}P < 0.01$ vs JSH-23 0 μM, n = 3. **B**. The mRNA levels of SPTBN1, SOCS1 and p65 were analyzed by QRT-PCR in SNU-449 cells transfected with p65 siRNA for 48 h. $^{**}P < 0.01$ vs siCtrl, n = 3.
